# Supplementary material for: Boosted high-throughput D⁺ transfer from D₂O to unsaturated bonds via Pdδ+ cathode for solvent-free deuteration
Source: Nat Commun. 2025 May 15;16:4503. doi: 10.1038/s41467-025-59776-1 (PMC12081598; doi:10.1038/s41467-025-59776-1)
Supplement: Supplementary file 1 — Supplementary Information [file 41467_2025_59776_MOESM1_ESM.pdf]

Boosted high-throughput D^+ transfer from pure D_2O to unsaturated bonds via a $Pd^{\delta+}$ cathode for solvent-free deuteration

Xiu-Feng Zhang[1], Shi-Nan Zhang[1], Zhao Zhang[1], Bing-Liang Leng[1], Kai-Yuan Lu[1], Jie-Sheng Chen[1], and Xin-Hao Li[1*]

[1]School of Chemistry and Chemical Engineering, Frontiers Science Center for Transformation Molecules, Shanghai Jiao Tong University, Shanghai 200240, P. R. China.

[*]Email: xinhaoli@sjtu.edu.cn;

Table of Contents

1 Figure S1-S18 4

Figure S1. Deuteration methodologies for deuterated compounds. 4

Figure S2. Digital photos the integrated all-solid reactor. 5

Figure S3. SEM images of $Pd^{\delta+}$/NC LDC. 6

Figure S4. TEM images of $Pd^{\delta+}$/NC. 7

Figure S5. TEM images of $Pd^{\delta+}$/NC. 8

Figure S6. XRD patterns of NC, Pd/C and $Pd^{\delta+}$/NC samples. 9

Figure S7. The ^{1}H-NMR (500 MHz, DMSO-d6) spectra of (a) cathode solution, (b) benzaldehyde and (c) benzyl alcohol. 10

Figure S8. EIS and DRT results of $Pd^{\delta+}$/NC LDC. 11

Figure S9. Nyquist plots and DRT results of the EIS for $Pd^{\delta+}$/NC LDC at different voltages with different ionomer content. 12

Figure S10. Nyquist plots and DRT results of the EIS for Pd/C LDC at different voltages with different ionomer content. 13

Figure S11. Resistance of (a) R_{D-T} and (b) R_K for $Pd^{\delta+}$/NC LDC and Pd/C LDC at different voltages. 14

Figure S12. *In situ* ATR-SEIRAS of (a) D_2O and (b) Benzenemethan-d_2-ol. 15

Figure S13. The corresponding optimal configuration of each step in deuterated reduction of benzaldehyde on the $Pd^{\delta+}$ surface. 16

Figure S14. Gibbs free energy diagrams of each step of the deuterated reduction of benzaldehyde on the Pd/C (grey line), $Pd^{\delta+}/N_{1.7}C$ (blue line) and $Pd^{\delta+}/N_{2.1}C$ (red line) surface and step-by-step adsorption configurations. 17

Figure S15. Bader charge analysis of Pd cluster on NC support in $Pd^{\delta+}/N_{2.1}C$ model. 18

Figure S16. Bader charge analysis of Pd cluster on NC support in $Pd^{\delta+}/N_{1.7}C$ model. 19

Figure S17. High-resolution (a) N *1s* and (b) C *1s* XPS spectra of Pd/C and $Pd^{\delta+}$/NC samples. 20

Figure S18. Digital photo of the device integrated $Pd^{\delta+}$/NC LDC into the membrane electrode assembly (MEA) electrolyzer. 21

Figure S19. Chronopotentiometry curves of the device with different current. 22

Figure S20. SEM images of $Pd^{\delta+}$/NC LDC. 23

Figure S21. HRTEM images of $Pd^{\delta+}$/NC. 24

Figure S22. XRD patterns of $Pd^{\delta+}$/N2.1C LDC sample. 25

Figure S23. High-resolution (a) Pd *3d* and (b) N *1s* XPS spectra of Pd/NC sample before (gray line) and after (red line) 500 h reaction. 26

Figure S24. Separation of deuterated water at the cathode. 27

Figure S25. XRD patterns of (a) GDL and IrO_2 (b) MEA before and after running the device for 32 h at 0.6 A. 28

2 Table S1-S6 29

Table S1. Elemental contents of samples obtained by XPS results. 29

Table S2. Element contents of $Pd^{\delta+}/N_{2.1}C$ sample estimated via ICP and EA. 30

Table S3. Substrate scope of $Pd^{\delta+}$/NC LDC in the deuterated reduction of aldehydes. 31

Table S4. Substrate scope of $Pd^{\delta+}$/NC LDC in the deuterated reduction of ketones. 32

Table S5. Substrate scope of $Pd^{\delta+}$/NC LDC in the deuterated reduction of olefins. 33

Table S6. Substrate scope of $Pd^{\delta+}$/NC LDC in the deuterated reduction of imines. 34

3 References 35

1 Figure S1-S18

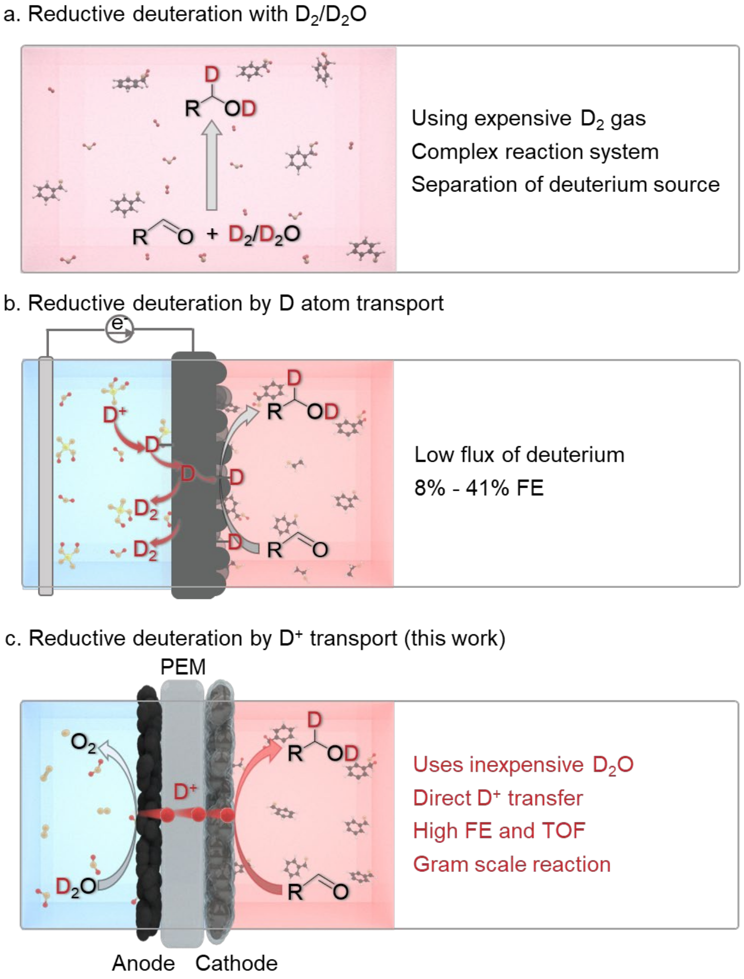

Figure S1. Deuteration methodologies for deuterated compounds. The figure illustrates the advantages and disadvantages of known (a) deuteration methods using D_2 and D_2O, (b) D atom transport, and (c) the schematic diagram of reductive deuteration via D^+ transport (this work).

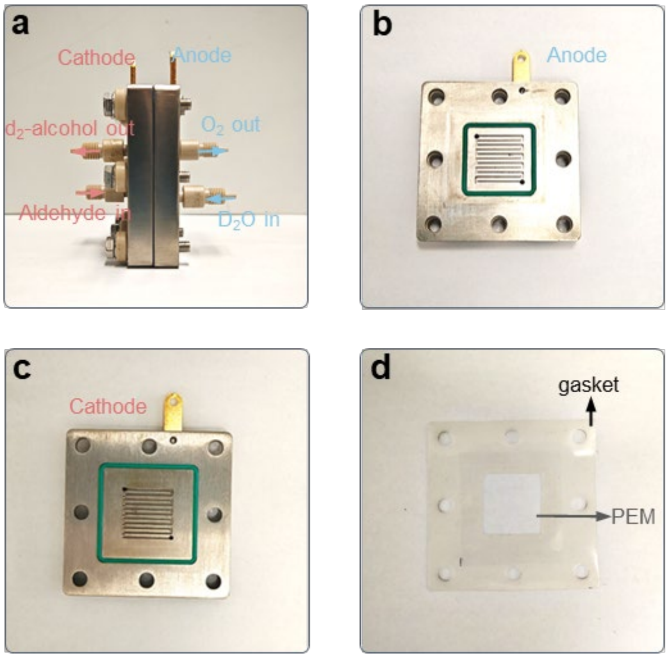

Figure S2. Digital photos the integrated all-solid reactor.

(a) The side view of the reactor. (b and c) The top view of anode and cathode plates. (d) The top view of the membrane and gasket.

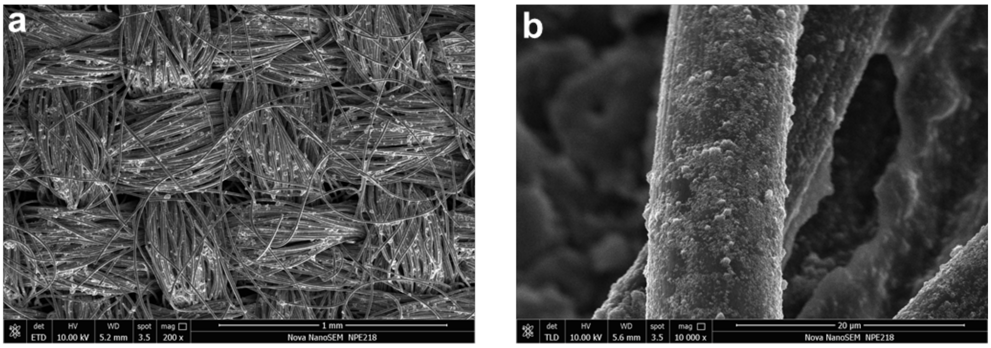

Figure S3. SEM images of Pd$^{\delta+}$/NC LDC. (a) Low magnification and (b) High-magnification SEM image of the Pd$^{\delta+}$/NC LDC. The Pd$^{\delta+}$/NC is uniformly loaded on a 4 cm^2 carbon cloth with a loading capacity of 1 mg cm^{-2}.

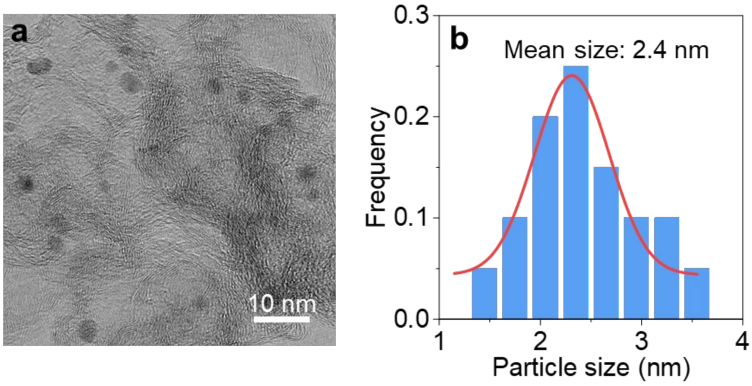

Figure S4. TEM images of Pd$^{\delta+}$/NC. (a) TEM image of Pd$^{\delta+}$/NC. (b) The size distribution of Pd nanoparticles. The mean size of Pd nanoparticles was 2.4 nm in this sample.

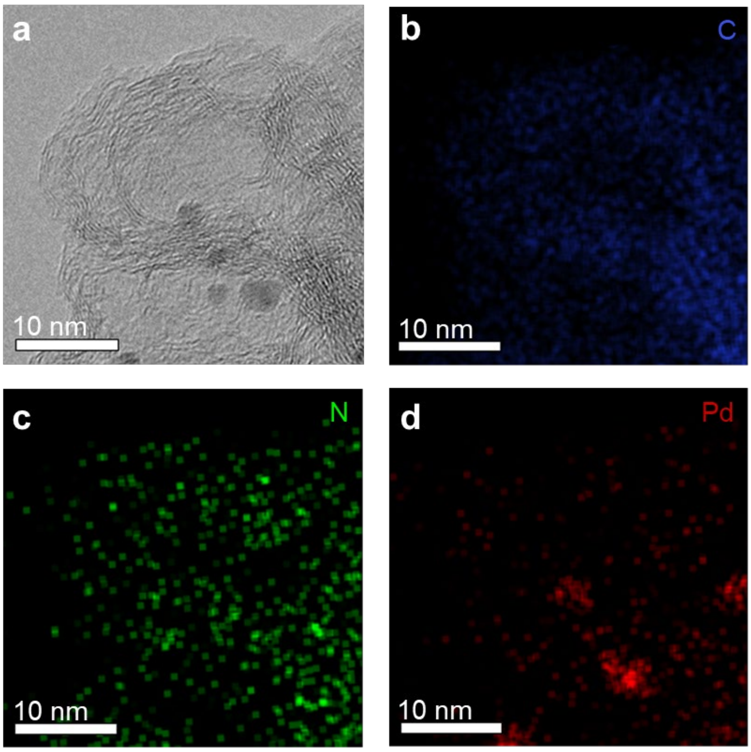

Figure S5. TEM images of Pd$^{\delta+}$/NC. (a) TEM image of Pd$^{\delta+}$/NC. (b-d) Element mapping of C, N and Pd.

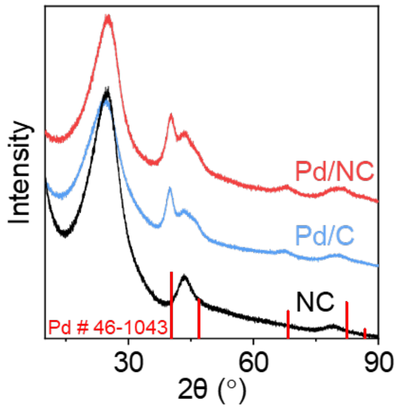

Figure S6. XRD patterns of NC, Pd/C and $Pd^{\delta+}$/NC samples. The characteristic sharp peaks were attributed to Pd with PDF # 06-1043.

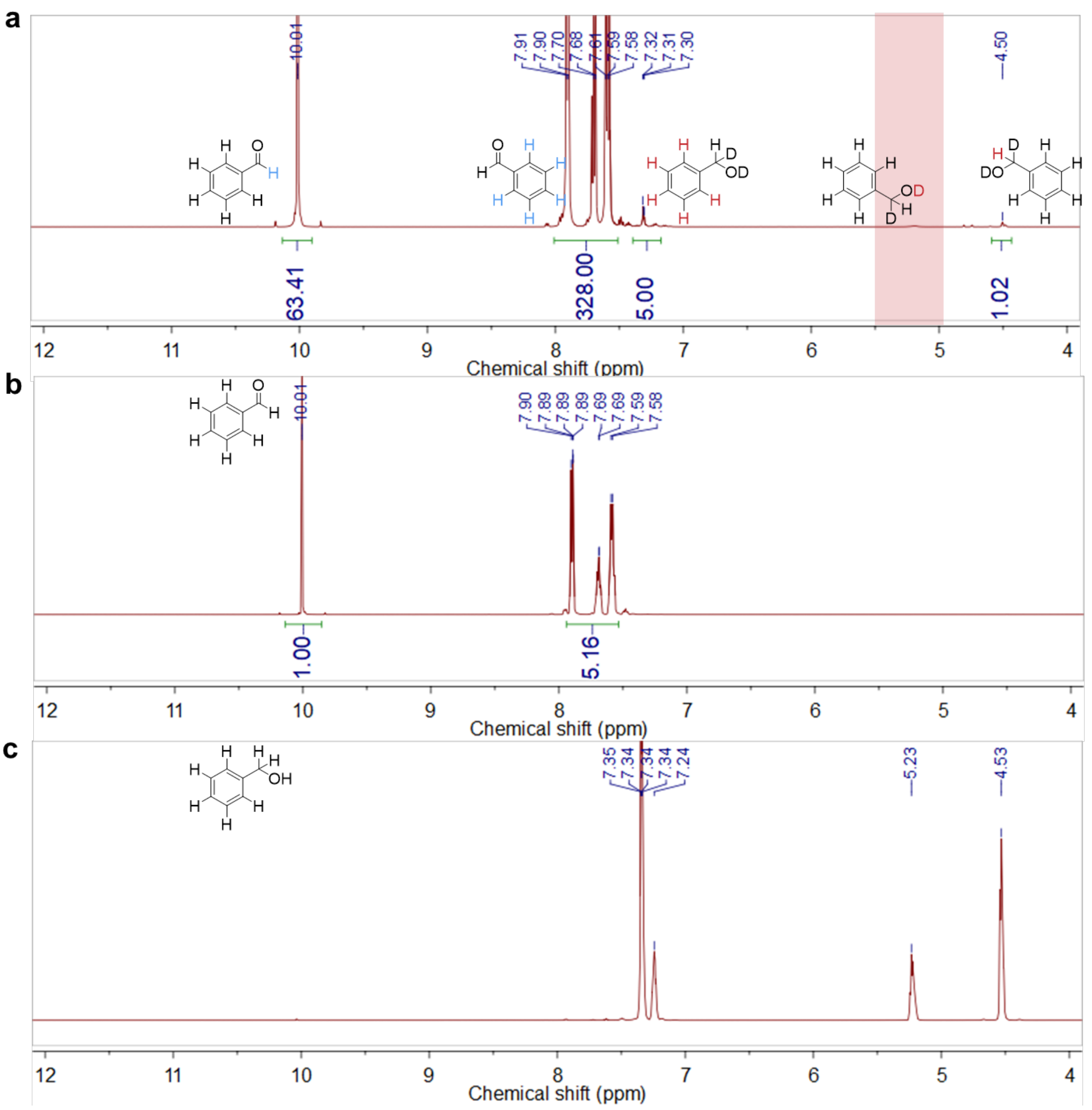

Figure S7. The ^{1}H-NMR (500 MHz, DMSO-d6) spectra of (a) cathode solution, (b) benzaldehyde and (c) benzyl alcohol.

The NMR data demonstrated the formation of deuterated benzyl alcohol as the only detectable product. Based on above analytic results in Figure S5. The spectrum of electrochemical reductive deuteration product, benzenemethan-d-ol-d, was analyzed as follows. The hydrogen peak at 4.50 ppm corresponded to hydrogen of -CHD- and was integrated into 0.99. This residual signal is derived from -CHO of benzaldehyde. The D-incorporation of this site is:

$$\text{Deuterium incorporation} = \left(\frac{deterium\ atom}{number\ of\ labelling\ sites}\right) \times 100\%$$

$$= \left(\frac{2 - 1.02}{1}\right) \times 100\%$$

$$= 98\%$$

The hydrogen peak at 7.30-7.32 ppm corresponded to the hydrogens of aromatic ring.

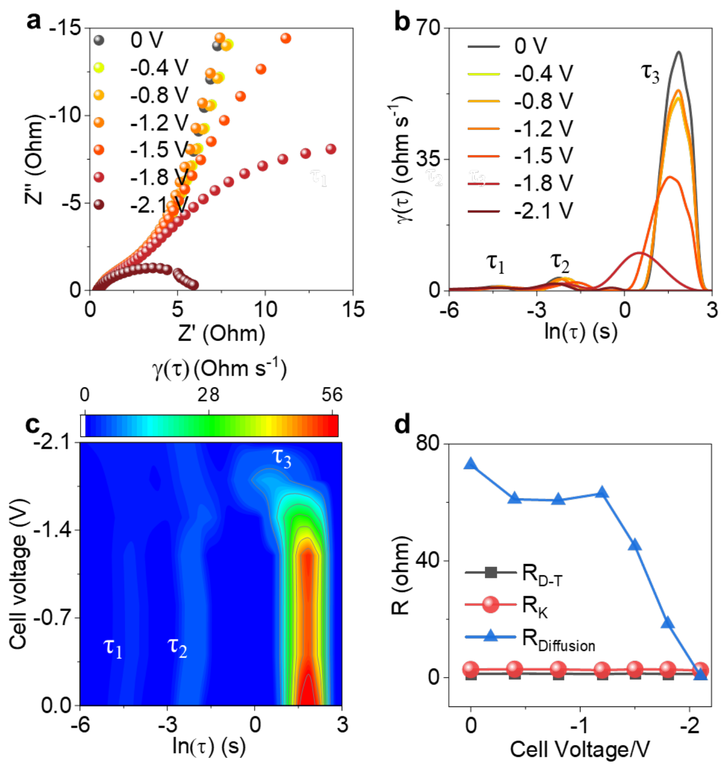

Figure S8. EIS and DRT results of Pd$^{\delta+}$/NC LDC. (a) Nyquist plots of the EIS for Pd$^{\delta+}$/NC LDC at different voltages. (b-c) The corresponding DRT result of EIS. (d) Resistance of Pd$^{\delta+}$/NC LDC at different voltages.

DRT analysis of the EIS data of Pd/NC at 0 V revealed three main peaks, τ_1 (within the region of -6 to -3), τ_2 (within the region of -3 to 0) and τ_3 (within the region of 0 to 3). Generally, due to the inverse relationship between τ and *f*, the high-frequency τ1 is considered to correspond to the ion (D^+) transfer resistance ($R_{D\text{-}T}$), while the mid-frequency τ2 is associated with kinetic-related resistance (R_K), and the low-frequency τ3 is attributed to the diffusion impedance ($R_{Diffusion}$). [1-4] As the voltage increases, the $R_{Diffusion}$ rapidly decreases, and when the cell voltage reaches practical conditions (around 2.1 V), the diffusion impedance drops below $R_{D\text{-}T}$ and R_K. Thus, the catalytic enhancement of Pd$^{\delta+}$/NC LDC for the deuteration of benzaldehyde primarily stems from its ability to lower the $R_{D\text{-}T}$ and R_K.

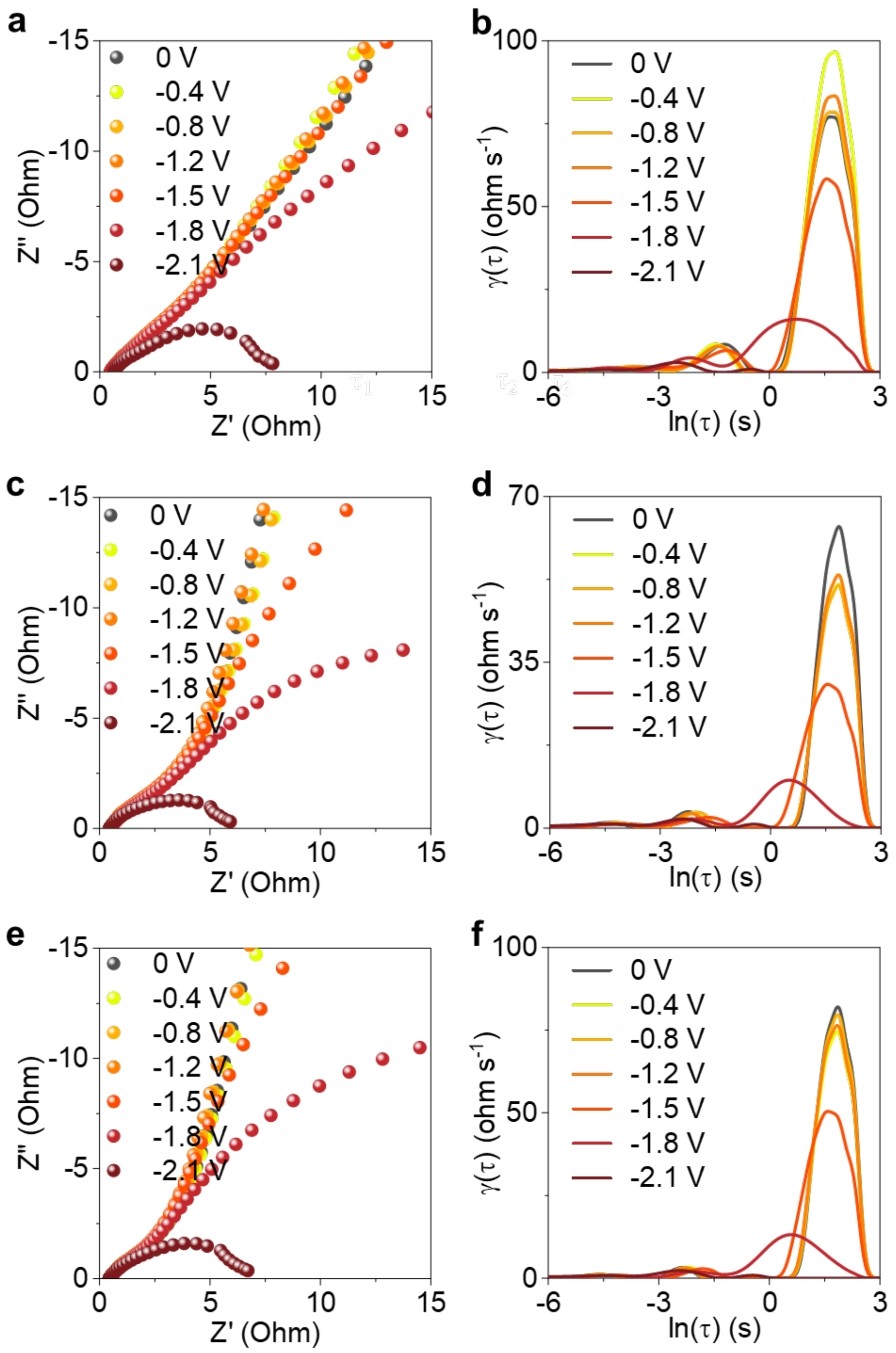

Figure S9. Nyquist plots and DRT results of the EIS for $Pd^{\delta+}$/NC LDC at different voltages with different ionomer content. The ionomer content of $Pd^{\delta+}$/NC LDC were (a-b) 0.56 mg cm^{-2}, (c-d) 2.1 mg cm^{-2} and (e-f) 4.5 mg cm^{-2}.

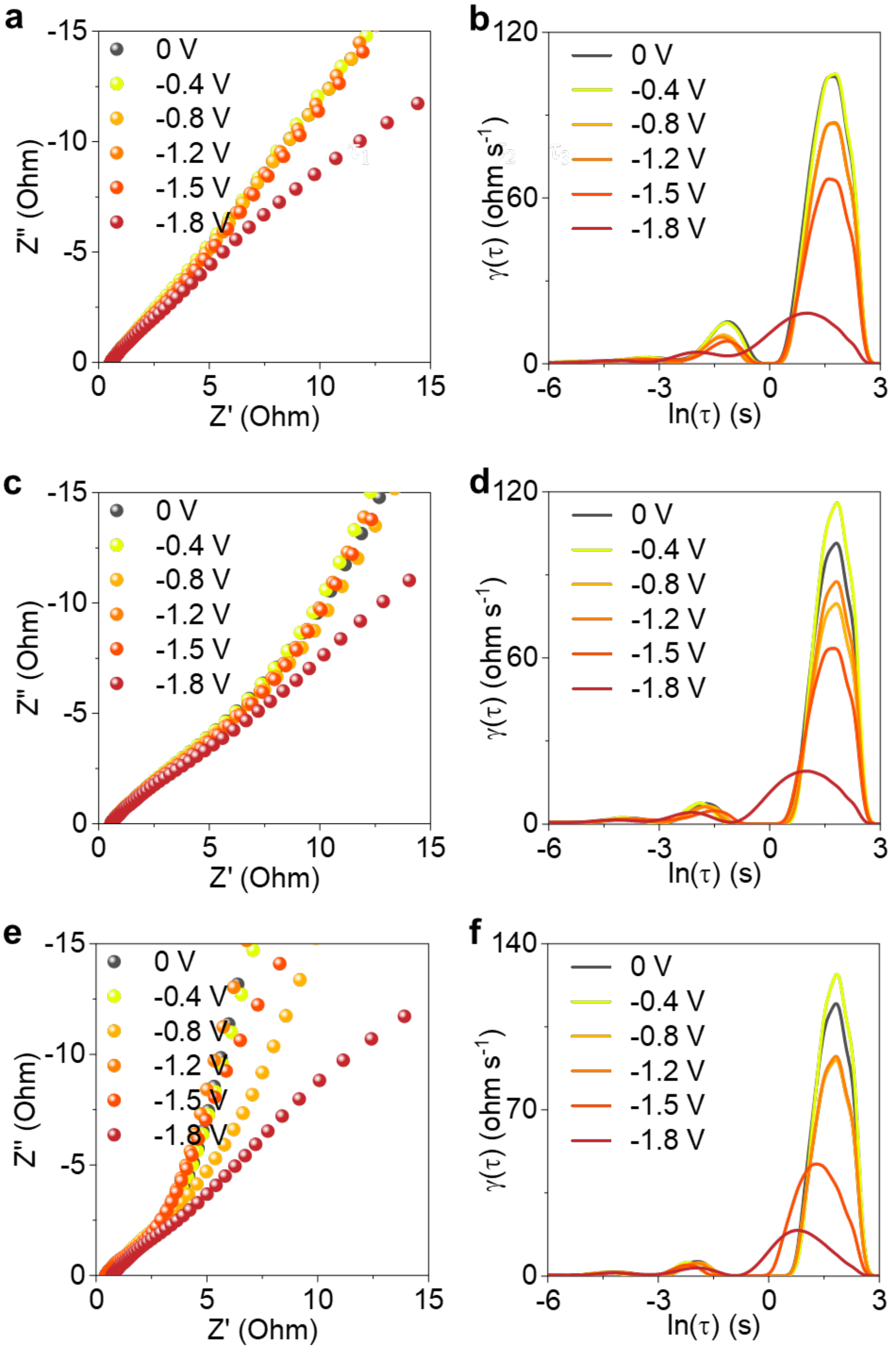

Figure S10. Nyquist plots and DRT results of the EIS for Pd/C LDC at different voltages with different ionomer content. The ionomer content of Pd/C LDC were (a-b) 0.56 mg cm^{-2}, (c-d) 2.1 mg cm^{-2} and (e-f) 4.5 mg cm^{-2}.

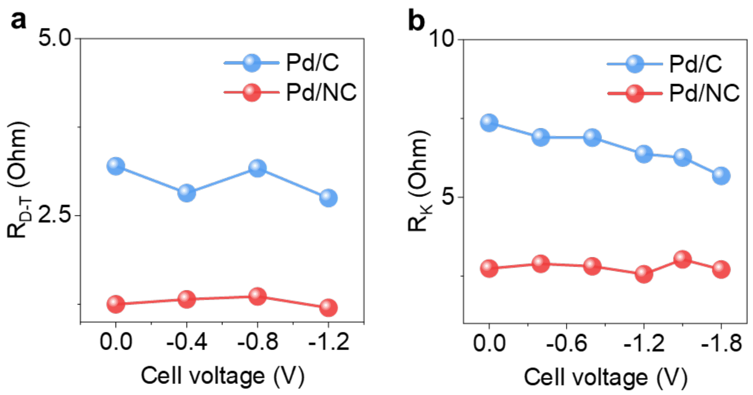

Figure S11. Resistance of (a) R_{D-T} and (b) R_K for $Pd^{\delta+}$/NC LDC and Pd/C LDC at different voltages.

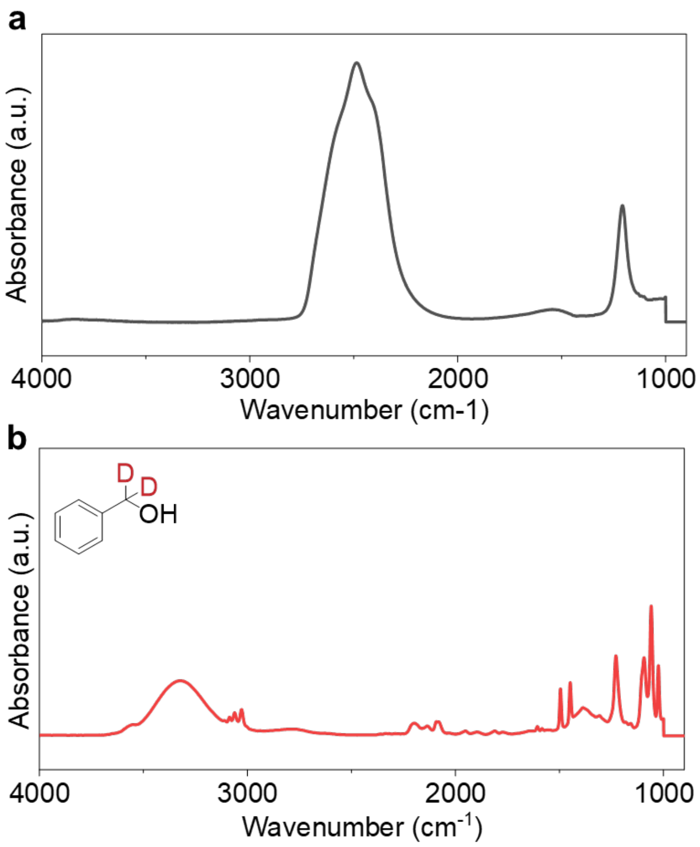

Figure S12. ***In situ*** **ATR-SEIRAS of (a) D_2O and (b) Benzenemethan-d$_2$-ol.**

The infrared peaks of the -O-D bond are primarily located at 2493 cm^{-1} and 1210 cm^{-1},[5] and the infrared peaks of the benzenemethan-d$_2$-ol are primarily located at 3319 cm^{-1}, 1495 cm^{-1}, 1448 cm^{-1}, 1228 cm^{-1}, 1058 cm^{-1}. The main peaks of benzenemethan-d-ol-d (the product of this work) are consistent with those of benzenemethan-d$_2$-ol, except for the -O-H vibration peak at 3315 cm^{-1}, which is absent in benzenemethan-d-ol-d (due to the deuteration of -O-H in benzenemethan-d-ol-d). The -C-H peak in benzenemethan-d-ol-d and benzaldehyde, are consistent with those reported in the literature.[6]

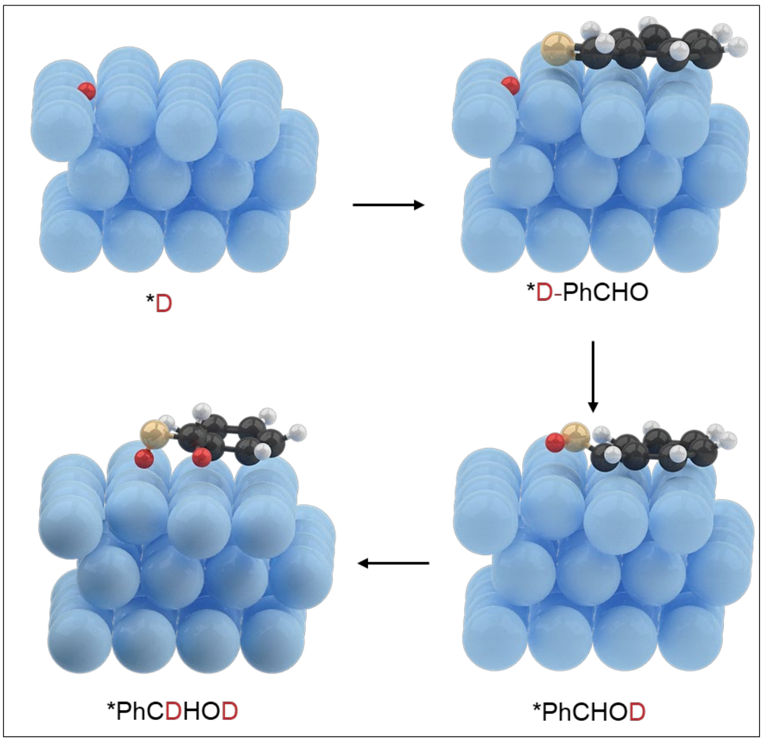

Figure S13. The corresponding optimal configuration of each step in deuterated reduction of benzaldehyde on the $Pd^{\delta+}$ surface. Blue, Pd; black, C; orange, O; red, D; white, H.

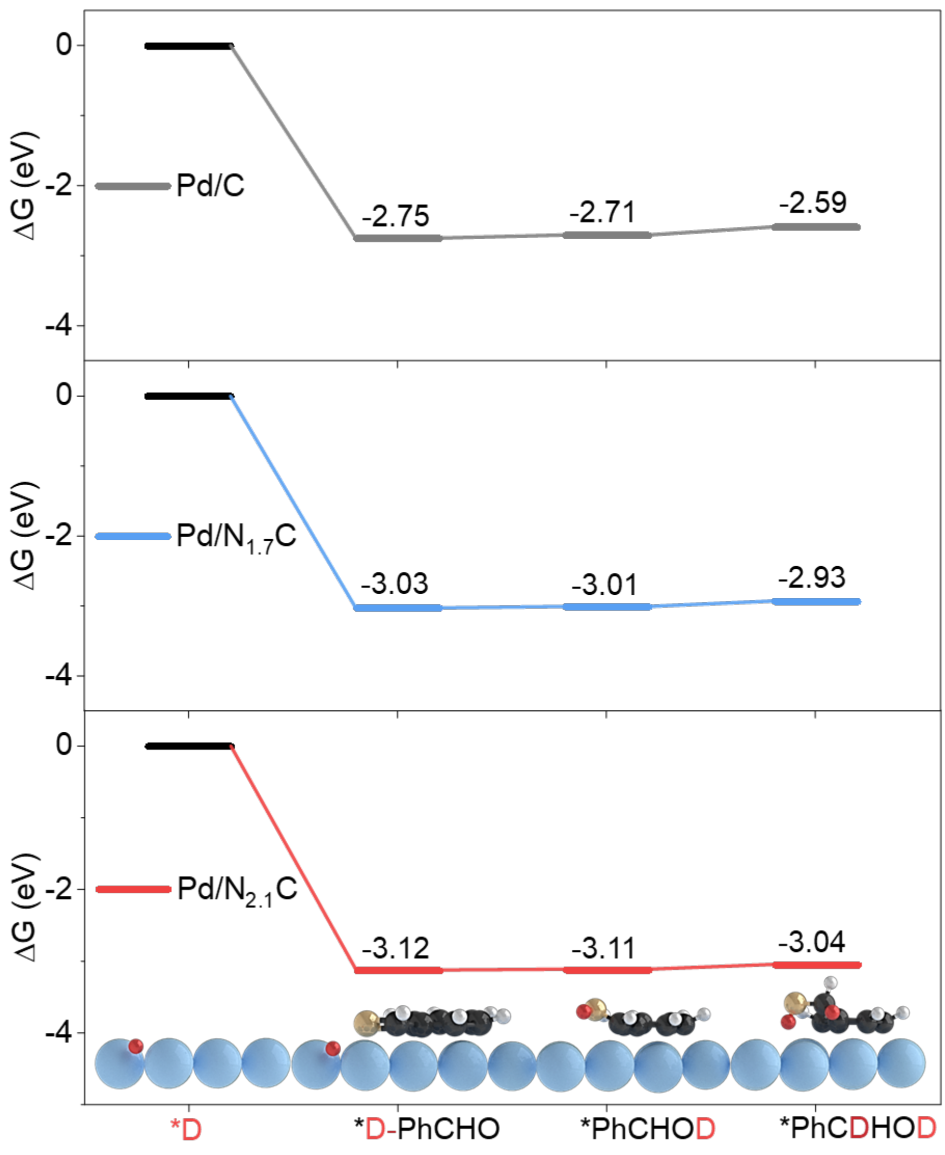

Figure S14. Gibbs free energy diagrams of each step of the deuterated reduction of benzaldehyde on the Pd/C (grey line), $Pd^{\delta+}/N_{1.7}C$ (blue line) and $Pd^{\delta+}/N_{2.1}C$ (red line) surface and step-by-step adsorption configurations. Blue, Pd; black, C; orange, O; red, D; white, H.

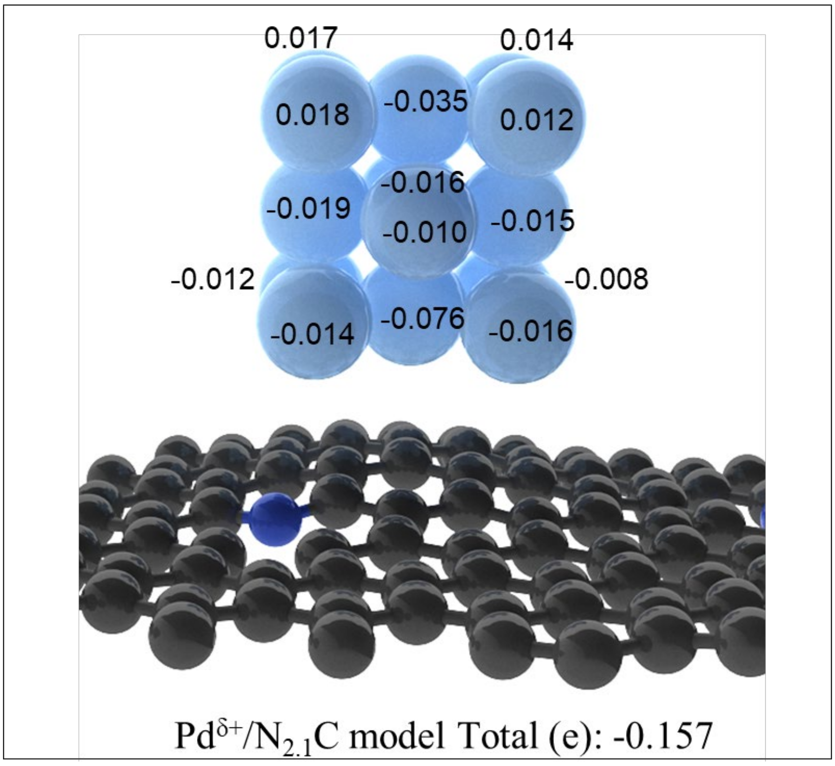

Figure S15. Bader charge analysis of Pd cluster on NC support in Pd$^{\delta+}$/N$_{2.1}$C model. Light blue, Pd; black, C; deep blue, N.

We quantify charge exchange between the Pd cluster and the NC support model. The Pd transferred 0.157 electrons to the NC support model. Therefore, 0.011 electrons flow from each Pd atom to NC support model via the rectifying contact, calculated based on the number of Pd atoms.

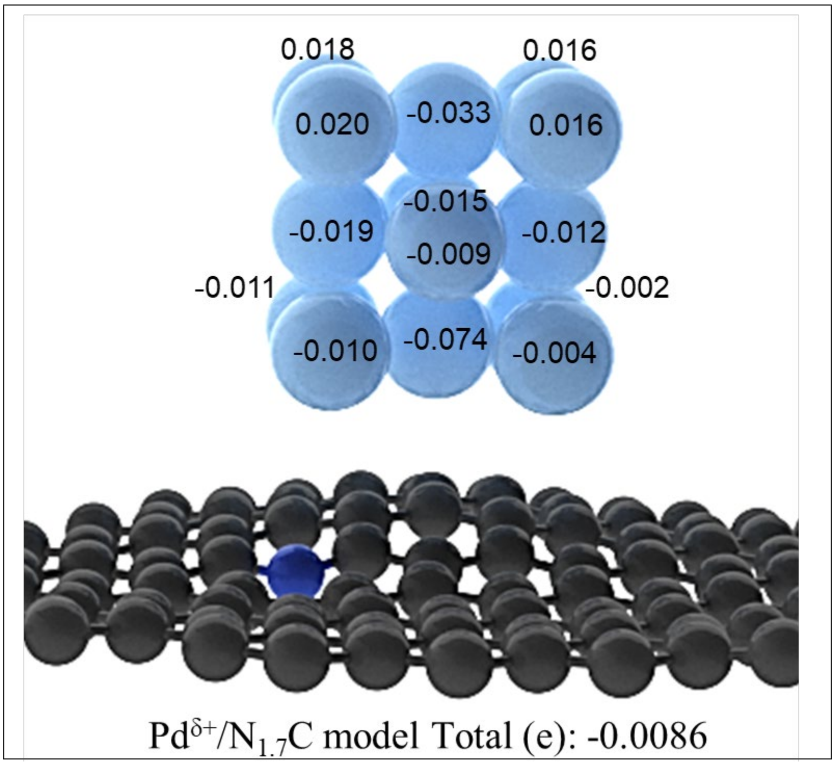

Figure S16. Bader charge analysis of Pd cluster on NC support in $Pd^{\delta+}/N_{1.7}C$ model. Light blue, Pd; black, C; deep blue, N.

We quantify charge exchange between the Pd cluster and the NC support model. The Pd transferred 0.120 electrons to the NC support model. Therefore, 0.009 electrons flow from each Pd atom to NC support model via the rectifying contact, calculated based on the number of Pd atoms.

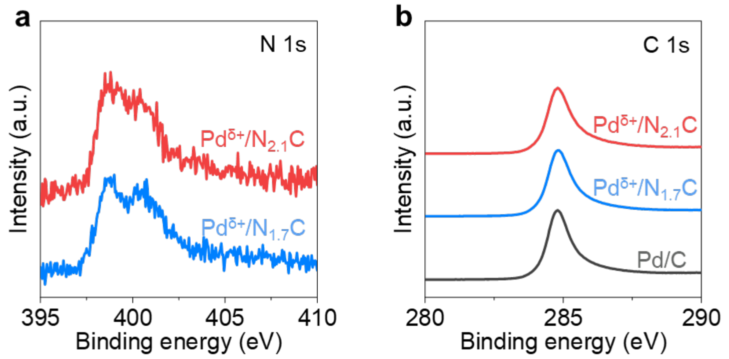

Figure S17. High-resolution (a) N *1s* and (b) C *1s* XPS spectra of Pd/C and Pd$^{\delta+}$/NC samples. N in Pd$^{\delta+}$/N$_{1.7}$C and Pd$^{\delta+}$/N$_{2.1}$C were mainly pyridinic N and pyrrolic N.

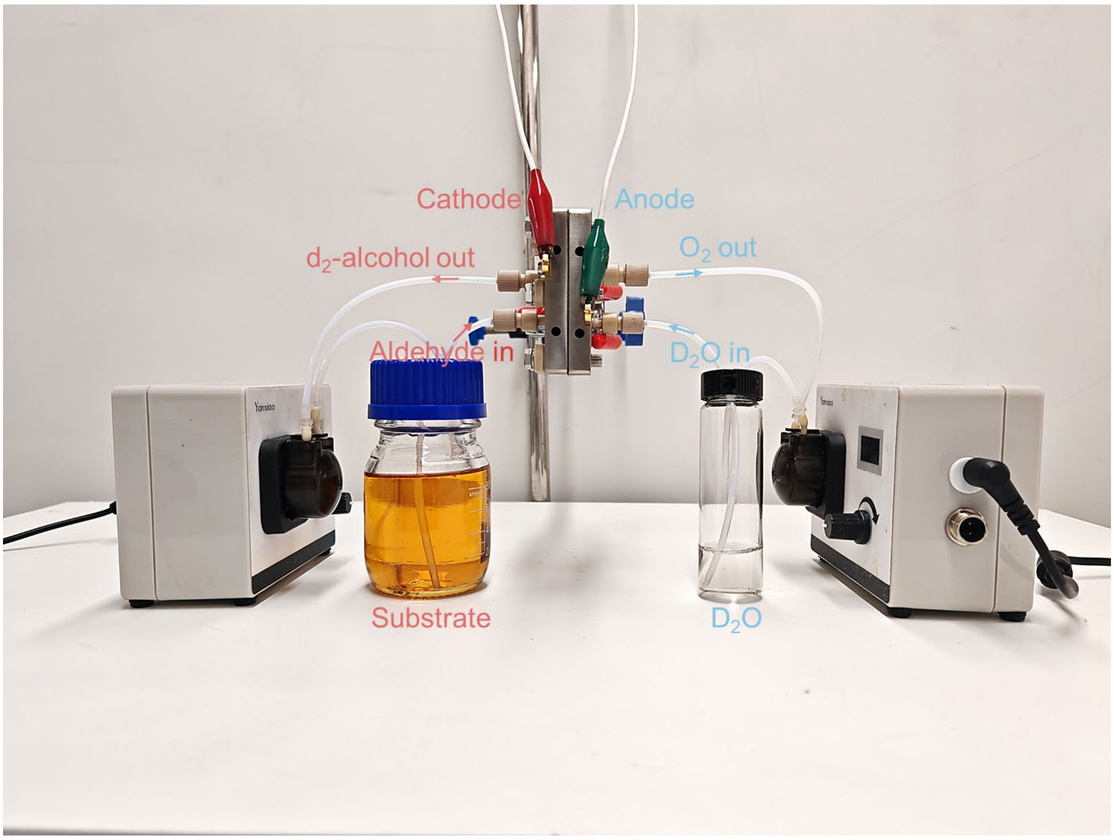

Figure S18. Digital photo of the device integrated Pd$^{\delta+}$/NC LDC into the membrane electrode assembly (MEA) electrolyzer.

This device used commercially available GDL (Pt coated titanium felt, SP-PTL, Hefei Conservation of Momentum Green Energy Co., Ltd.) and IrO_2 MEA (Ir loading: 0.75mg cm^{-2}, Hefei Conservation of Momentum Green Energy Co., Ltd.). The anode side was circulated with pure D_2O at 1 mL min^{-1}. The cathode side was circulated with 1 mol pure benzaldehyde at 1 mL min^{-1}. The generated cathode products were analyzed by GC-MS to determine the components of products and calculate the conversion and selectivity. For D_2O at the anode, since it is not in direct contact with the substrate, it can be reused after the reaction without separation.

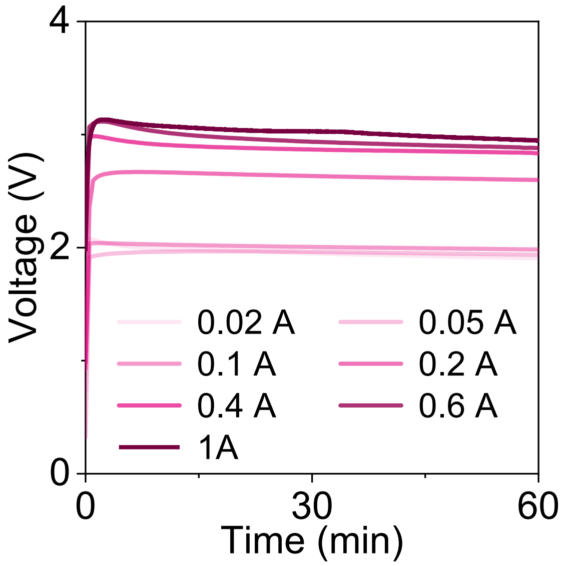

Figure S19. Chronopotentiometry curves of the device with different current.

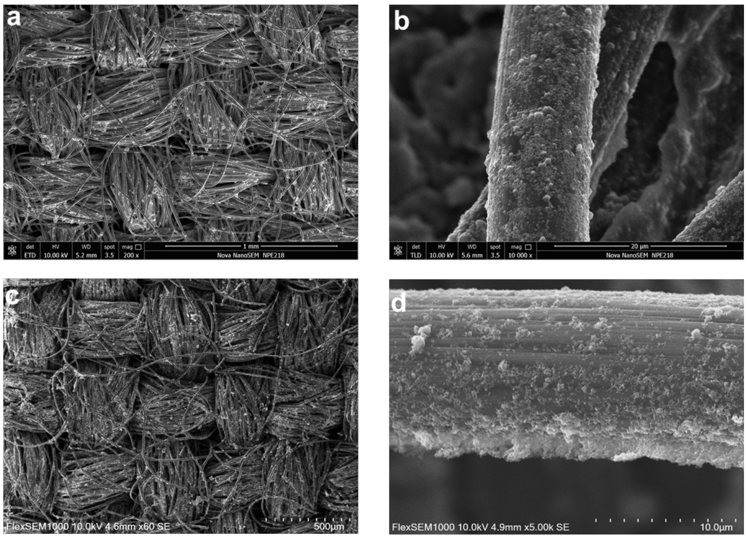

Figure S20. SEM images of $Pd^{\delta+}$/NC LDC. The microstructure of the $Pd^{\delta+}$/NC LDC remained well stable before (a-b) and after (c-d) the 500-hour reaction at 20 mA.

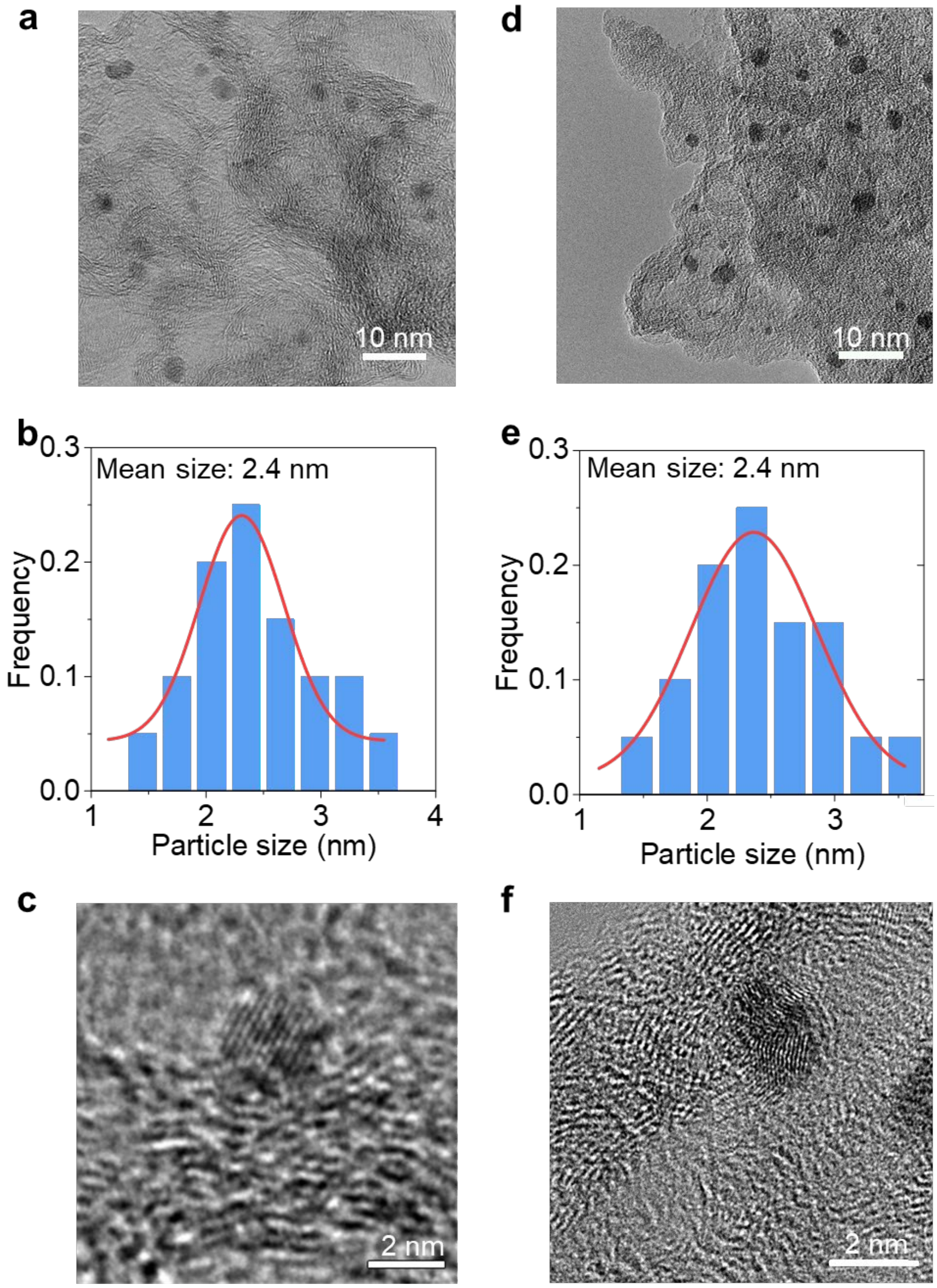

Figure S21. HRTEM images of Pd$^{\delta+}$/NC. The microstructure of Pd particles and carbon spheres has been well maintained before (a-c) and after (d-f) deuteration of benzaldehyde to benzyl alcohol over 500 h at 20 mA.

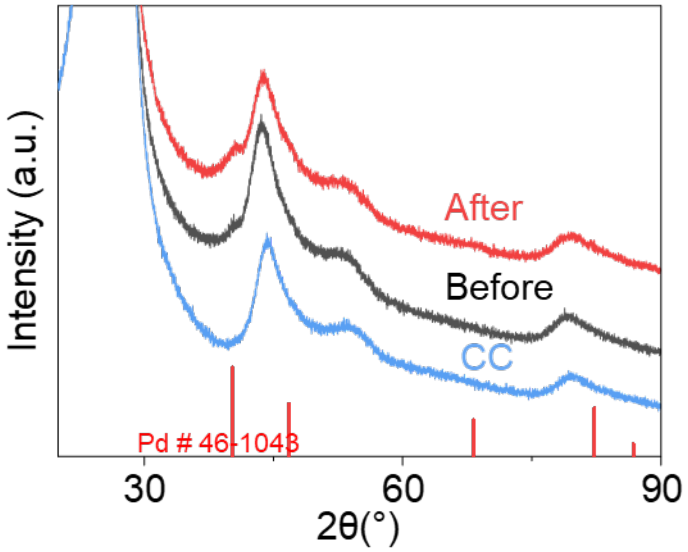

Figure S22. XRD patterns of $Pd^{\delta+}$/N2.1C LDC sample. The characteristic sharp peaks were attributed to Pd with PDF # 06-1043. The peak belonging to the carbon cloth in LDC at 44.7°, 53.8° and 79.9° did not change, while the peak belonging to the Pd (100) at 40.1° was still observed, despite the low Pd loading (3 wt%), after 500 h reaction.

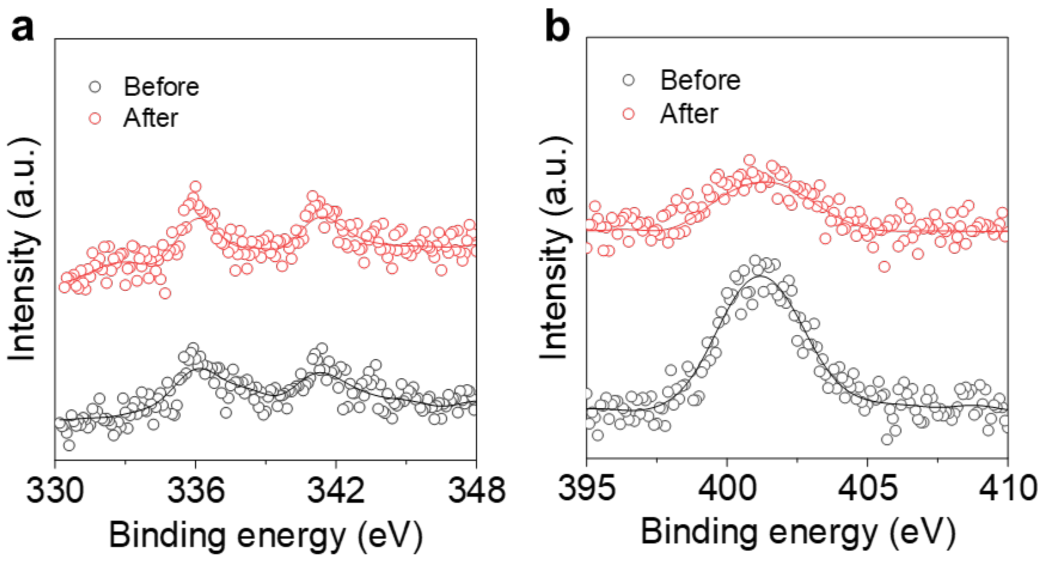

Figure S23. High-resolution (a) Pd *3d* and (b) N *1s* XPS spectra of Pd/NC sample before (gray line) and after (red line) 500 h reaction.

The peak positions of XPS of Pd and N did not change significantly, confirming the stability of Pd/NC catalyst before and after the reaction.

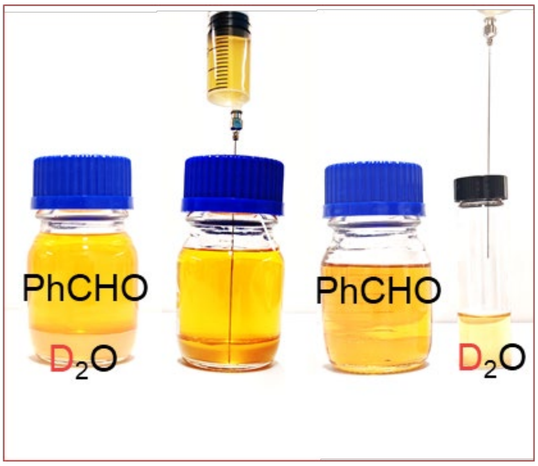

Figure S24. Separation of deuterated water at the cathode.

Deuterium water crossed the proton exchange membrane about 18 mL after the reaction at 600 mA for 20 h. Since D_2O is not mutually soluble with the substrate, D_2O was recovered from the anode liquid after reaction by simple liquid-liquid separation, and we obtained 17.8 mL of D_2O with a recovery rate above 98.8%.

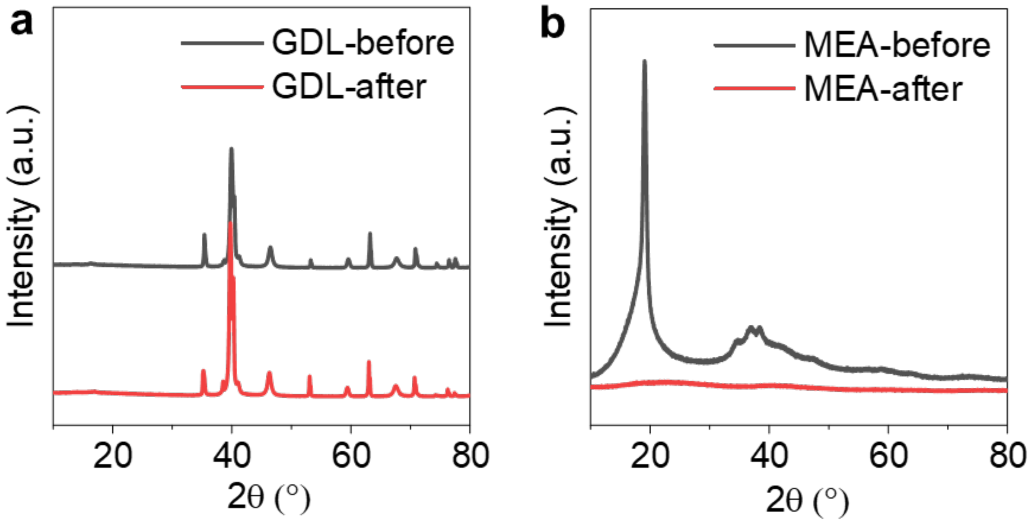

Figure S25. XRD patterns of (a) GDL and IrO_2 (b) MEA before and after running the device for 32 h at 0.6 A.

After running the device at 0.6 A for 32 h, the XRD diffraction peak of GDL (Pt coated titanium felt) did not change significantly, but the XRD diffraction peak of InO_2 MEA disappeared, indicating that its stability was insufficient.

2 Table S1-S6

Table S1. Elemental contents of samples obtained by XPS results.

Catalysts	N contents (at. %)	C contents (at. %)	O contents (at. %)	Pd contents (at. %)
Pd/ C	0.2	97.8	1.8	0.28
$Pd^{\delta+}/N_{1.7}C$	1.7	95.8	2.2	0.39
$Pd^{\delta+}/N_{2.1}C$	2.1	95.7	1.9	0.45

Table S2. Element contents of $Pd^{\delta+}/N_{2.1}C$ sample estimated via ICP and EA.

	Pd contents (wt. %)	C contents (wt. %)	N contents (wt. %)
ICP	3.39	-	-
EA	-	96.6	3.4

Table S3. Substrate scope of $Pd^{\delta+}$/NC LDC in the deuterated reduction of aldehydes.

Entry	Reactant	Product	TOF (h^{-1})	FE (%)	Selectivity (%)
1	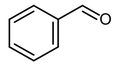	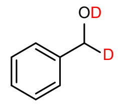	211	72	99
2	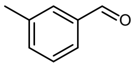	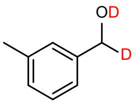	163	56	99
3	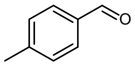	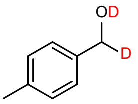	183	62	99
4	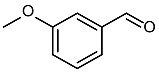	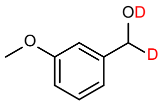	131	45	99
5	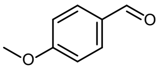	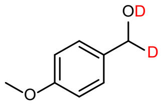	128	43	99
6	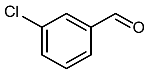	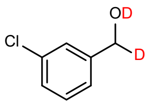	238	81	88
		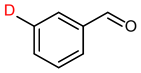	30	10	11
7	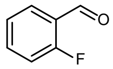	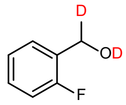	190	65	99
8	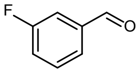	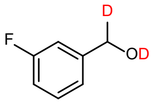	187	64	99
9	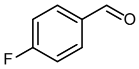	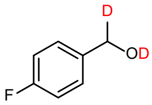	165	56	99

Reaction condition: 2 mL pure D_2O at 1 mL min^{-1} for anode and 2 mL pure reactant at 1 mL min^{-1} for cathode side. $Pd^{\delta+}$/NC LDC (1 mg cm^{-2}; 2 h) and RuO_2 anode at 20 mA. The generated cathode products were analyzed by GC-MS.

The by-product of entry 6 arises from dehalogenation of the substrate.

The currently reported bench-marked contactless reactor for deuterium transfer (Pd/Pd membrane reactor of Ref. 18) has only 7% FE for the reductive deuteration of benzaldehyde.

Table S4. Substrate scope of $Pd^{\delta+}$/NC LDC in the deuterated reduction of ketones.

Entry	Reactant	Product	TOF (h^{-1})	FE (%)	Selectivity (%)
1	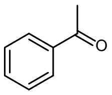	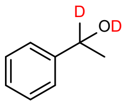	163	55	99
2	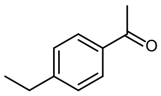	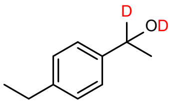	140	48	99
3	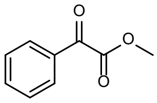	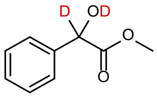	92	31	99
4	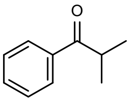	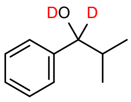	255	90	99

Reaction condition: 2 mL pure D_2O at 1 mL min^{-1} for anode and 2 mL pure reactant at 1 mL min^{-1} for cathode side. $Pd^{\delta+}$/NC LDC (1 mg cm^{-2}; 2 h) and RuO_2 anode at 20 mA. The generated cathode products were analyzed by GC-MS.

This is the first reductive deuteration of ketones achieved by contactless reactor for deuterium transfer.

Table S5. Substrate scope of $Pd^{\delta+}$/NC LDC in the deuterated reduction of olefins.

Entry	Reactant	Product	TOF (h^{-1})	FE (%)	Selectivity (%)
1	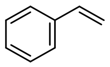	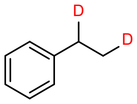	217	74	99
2	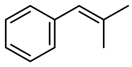	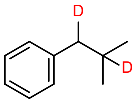	248	84	99
3	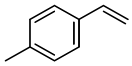	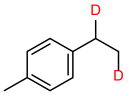	287	98	99
4	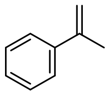	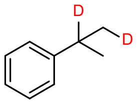	275	94	99
5	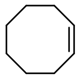	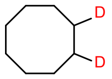	224	76	99
6	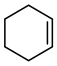	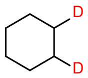	81	55	99

Reaction condition: 2 mL pure D_2O at 1 mL min^{-1} for anode and 2 mL pure reactant at 1 mL min^{-1} for cathode side. $Pd^{\delta+}$/NC LDC (1 mg cm^{-2}; 2 h) and RuO_2 anode at 20 mA. The generated cathode products were analyzed by GC-MS.

The currently reported bench-marked contactless reactor for deuterium transfer (Pd/Pd membrane reactor of Ref. 18) has only 40% FE for the synthesis of deuterated alkanes.

Table S6. Substrate scope of $Pd^{\delta+}$/NC LDC in the deuterated reduction of imines.

Entry	Reactant	Product	TOF (h^{-1})	FE (%)	Selectivity (%)
1	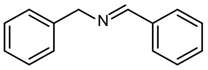	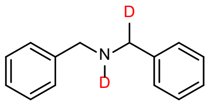	118	40	99
2	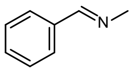	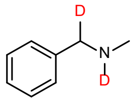	32	11	99

Reaction condition: 2 mL pure D_2O at 1 mL min^{-1} for anode and 2 mL pure reactant at 1 mL min^{-1} for cathode side. $Pd^{\delta+}$/NC LDC (1 mg cm^{-2}; 2 h) and RuO_2 anode at 20 mA. The generated cathode products were analyzed by GC-MS.

The currently reported bench-marked contactless reactor for deuterium transfer (Pd/Pd membrane reactor of Ref. 18) has only 8% FE for the reductive deuteration of imines.

3 References

1 Chen, J., Quattrocchi, E., Ciucci, F. & Chen, Y. Charging processes in lithium-oxygen batteries unraveled through the lens of the distribution of relaxation times. *Chem.* **9**, 2267-2281 (2023).

2 Giesbrecht, P. K. & Freund, M. S. Investigation of Water Oxidation at IrO2 Electrodes in Nafion-Based Membrane Electrode Assemblies Using Impedance Spectroscopy and Distribution of Relaxation Times Analysis. *The Journal of Physical Chemistry C* **126**, 17844-17861 (2022).

3 Müller-Hülstede, J. *et al.* What determines the stability of Fe-N-C catalysts in HT-PEMFCs? *International Journal of Hydrogen Energy* **50**, 921-930 (2024).

4 Soni, R. et al. Lithium-sulfur battery diagnostics through distribution of relaxation times analysis. *Energy Storage Materials* **51**, 97-107 (2022).

5 Wu, Y. et al. Electrosynthesis of a nylon-6 precursor from cyclohexanone and nitrite under ambient conditions. *Nature Communications* **14** (2023).

6 Zhang, Z. et al. Electrocatalytic Aromatic Alcohols Splitting to Aldehydes and H(2) Gas. *J. Am. Chem. Soc.* **146**, 27179-27185 (2024).
