# Supplementary material for: Boosted high-throughput D⁺ transfer from D₂O to unsaturated bonds via Pdδ+ cathode for solvent-free deuteration
Source: Nat Commun. 2025 May 15;16:4503. doi: 10.1038/s41467-025-59776-1 (PMC12081598; doi:10.1038/s41467-025-59776-1)
Supplement: Supplementary file 2 — Description Of Additional Supplementary File [file 41467_2025_59776_MOESM2_ESM.pdf]

Description of Additional supplementary files

Supplementary Data 1

Atomic coordinate information for the DFT calculated Pd/$N_{1.7}C$ model

Supplementary Data 2

Atomic coordinate information for the DFT calculated Pd/$N_{2.1}C$ model

Supplementary Data 3

Atomic coordinate information for the DFT calculated Pd-0.54e model

Supplementary Data 4

Atomic coordinate information for the DFT calculated Pd-0.41e model
