# Supplementary material for: Boosted high-throughput D⁺ transfer from D₂O to unsaturated bonds via Pdδ+ cathode for solvent-free deuteration
Source: Nat Commun. 2025 May 15;16:4503. doi: 10.1038/s41467-025-59776-1 (PMC12081598; doi:10.1038/s41467-025-59776-1)
Supplement: Supplementary file 3 — Supplementary Data 1 [file 41467_2025_59776_MOESM3_ESM.docx]

N5C

1.00000000000000

17.2199993133999989 0.0000000000000000 0.0000000000000000

-8.6099996566999994 14.9129568584999994 0.0000000000000000

0.0000000000000000 0.0000000000000000 20.0000000000000000

C N Pd

94 2 14

Direct

0.9969811061744434 0.0005616181581285 0.0893817186753217

0.0448180508049013 0.0942454770284532 0.0992417904918429

0.1399830493079345 -0.0015774621920846 0.0922977188646454

0.1875880104352560 0.0929103171668121 0.0991916854451210

0.2842750464735984 -0.0003557955023763 0.0898790196299921

0.3322067639729164 0.0953320116280058 0.0914579787042366

0.4263184969851929 -0.0000638582843960 0.0850410555706167

0.4740994449309991 0.0965902553450707 0.0833797278063714

0.5662521156715804 -0.0021651087554507 0.0788418894996648

0.6130628146344235 0.0937451817785380 0.0769465427342451

0.7073322649298340 -0.0003292464407210 0.0728825546821842

0.8557228120744933 0.0122232262280300 0.0812804637149895

0.9079732541828418 0.1142760560779801 0.0886477917263694

-0.0010499726325675 0.1439212795166838 0.1016091734181762

0.0497430604991288 0.2371778123584805 0.1157469868851830

0.1401027968391598 0.1404048972536306 0.1054029891304577

0.1888579121101709 0.2340056235827944 0.1167687259144412

0.2827573820468627 0.1402003184043824 0.0987604057832542

0.3301196913819386 0.2345609097083886 0.1040910947749488

0.4281122200348907 0.1459133972890667 0.0869291509595450

0.4759605795419629 0.2425556370787814 0.0858294358394344

0.5687711628741106 0.1457666686957384 0.0793973815548919

0.6184236428644504 0.2434443921672018 0.0781137313776157

0.7074481394138651 0.1355384294183748 0.0729513277641382

0.7607500668668858 0.2317675946579141 0.0763515907615285

0.9083465767484660 0.2199231003071006 0.0904428552614015

-0.0012906307694879 0.2802340238955644 0.1058282792762952

0.0456005060184300 0.3763710901832341 0.1066243804949650

0.1430994553041665 0.2830976175893833 0.1251000121300949

0.1913364781836616 0.3806935474089166 0.1281518981809818

0.2833932036409997 0.2812487598986505 0.1168768898846479

0.3330448299517416 0.3775700153176862 0.1214344019145138

0.4238149841370099 0.2853380689118771 0.0932689338005289

0.5725538945191024 0.2979869128062556 0.0804669611964668

0.6252234253699059 0.3987984340157055 0.0793578464666632

0.7144512578660976 0.2834803726493451 0.0769330820708527

0.7631024851178326 0.3785759775656117 0.0776112544854750

0.8561075821602442 0.2702698632888655 0.0828721437508602

0.9038404947413318 0.3703228201982615 0.0849673794837869

-0.0014789813249882 0.4228360325938263 0.0935437239643148

0.0473698841381974 0.5194555029749656 0.0915099882592042

0.1413170173520638 0.4266687936174404 0.1162976548600952

0.1906906363828682 0.5215779541724059 0.1052956479224102

0.2872399007529595 0.4281424348107984 0.1239611128814962

0.3364062151486310 0.5227891829227572 0.1116327625964301

0.4257409153856790 0.4211368303731766 0.1020773361999314

0.4778400828641173 0.5170106867679914 0.0950774130810171

0.6251267207681864 0.5074697314989701 0.0801192126341894

0.7181614369812425 0.4302141510479086 0.0773321459333618

0.7697246583878825 0.5248599455052861 0.0764371055949981

0.8583488042416026 0.4223680136936068 0.0801080472200171

0.9072404436351977 0.5193498656996378 0.0805770948575521

0.0013861023871934 0.5681953674896710 0.0851600564200635

0.0481702018057497 0.6645231068257801 0.0853883585735055

0.1427468689092179 0.5683044566553624 0.0960514526681545

0.1900108810167767 0.6638293129756079 0.0927041427944702

0.2864534645896804 0.5692157097256505 0.1046150963418454

0.3333143316557498 0.6640091190583299 0.0961858207877738

0.4320427036065853 0.5672588367783010 0.0992133623476372

0.4776140296315962 0.6628258718561503 0.0906199384567830

0.5731646179984404 0.5567208425801408 0.0843311225967012

0.6192344684808591 0.6572944597819074 0.0809924065857634

0.7180514719897374 0.5681879076187607 0.0771545005275565

0.7628102819381749 0.6643897963530476 0.0769316243751826

0.8621582465568545 0.5705054872260195 0.0777642487068622

0.9071899568511522 0.6666572136008266 0.0788625373272454

0.0013322305046426 0.7137065093707406 0.0824886011529103

0.0472181638917625 0.8090934865186006 0.0845635100289464

0.1426577910960783 0.7121574696618831 0.0888753533081688

0.1895499438184556 0.8079699784000796 0.0887103495245909

0.2851159242163876 0.7115888229274583 0.0931529070170256

0.3317557508127376 0.8070739042419710 0.0897385644184288

0.4288112500051977 0.7107701568183081 0.0911493631187940

0.4741923758174290 0.8058277971403157 0.0859322010005844

0.5723329846539335 0.7085834022400089 0.0829794849259748

0.6182641309399221 0.8061726802103464 0.0792605470409585

0.7142668831645406 0.7112112672194485 0.0776376400892463

0.7607782531330857 0.8091275481639332 0.0773162956299482

0.8581565883950245 0.7140606839140302 0.0777830570017349

0.9037197288482247 0.8105163802090054 0.0798751107020850

-0.0013464555523029 0.8568128713256064 0.0836543319695671

0.0448014998960040 0.9518268877040216 0.0883040867460796

0.1421277892741543 0.8562690892189985 0.0876950223713100

0.1889295915596284 0.9516500285682101 0.0900790746708037

0.2845424270249049 0.8557046052815009 0.0890337122052878

0.3314288866402142 0.9514326131058434 0.0879228581802559

0.4263666037539712 0.8544738519800524 0.0866197808419513

0.4719879698490546 0.9501370956280920 0.0834286196242971

0.5688069366725178 0.8531170906280126 0.0808373071092324

0.6130456515345756 0.9488445171649295 0.0774025655545397

0.7142008380825789 0.8585445592682465 0.0766495454944018

0.7601318069502436 0.9559647299510864 0.0756040972523648

0.8557959144073217 0.8601263242418661 0.0792215756217670

0.9020326072138738 0.9583075165777477 0.0829213704464339

0.4662252516499155 0.3748992317399883 0.0889562653908473

0.7501735270139637 0.0889982091837623 0.0684700319633169

0.0878578947891854 0.2416361382689332 0.2267396327082908

0.0868663244129372 0.3387890655229522 0.3257921365296372

0.2029250587105096 0.2581692299348555 0.3159833999318790

0.2336770032692097 0.4063683751322363 0.2332694860731882

0.3397518755076542 0.3348001063216095 0.2257824122935850

0.4129789528810986 0.4894900177139422 0.2879224397792507

0.0983033914231576 0.4397880279339305 0.2268532568011594

0.2223729918330228 0.5348930766588423 0.3125953354338799

0.3605602649198380 0.5888543113479483 0.2267526304690143

0.1407824191742807 0.2863554613441163 0.4265629030444918

0.2757663308144205 0.4241221319886275 0.3671989937015470

0.3715487725470151 0.3434628546575587 0.3549073420748651

0.1520590227635241 0.4507439581058980 0.4230461733964651

0.3842970211459105 0.6009209998562542 0.3602984367395221

0.00000000E+00 0.00000000E+00 0.00000000E+00

0.00000000E+00 0.00000000E+00 0.00000000E+00

0.00000000E+00 0.00000000E+00 0.00000000E+00

0.00000000E+00 0.00000000E+00 0.00000000E+00

0.00000000E+00 0.00000000E+00 0.00000000E+00

0.00000000E+00 0.00000000E+00 0.00000000E+00

0.00000000E+00 0.00000000E+00 0.00000000E+00

0.00000000E+00 0.00000000E+00 0.00000000E+00

0.00000000E+00 0.00000000E+00 0.00000000E+00

0.00000000E+00 0.00000000E+00 0.00000000E+00

0.00000000E+00 0.00000000E+00 0.00000000E+00

0.00000000E+00 0.00000000E+00 0.00000000E+00

0.00000000E+00 0.00000000E+00 0.00000000E+00

0.00000000E+00 0.00000000E+00 0.00000000E+00

0.00000000E+00 0.00000000E+00 0.00000000E+00

0.00000000E+00 0.00000000E+00 0.00000000E+00

0.00000000E+00 0.00000000E+00 0.00000000E+00

0.00000000E+00 0.00000000E+00 0.00000000E+00

0.00000000E+00 0.00000000E+00 0.00000000E+00

0.00000000E+00 0.00000000E+00 0.00000000E+00

0.00000000E+00 0.00000000E+00 0.00000000E+00

0.00000000E+00 0.00000000E+00 0.00000000E+00

0.00000000E+00 0.00000000E+00 0.00000000E+00

0.00000000E+00 0.00000000E+00 0.00000000E+00

0.00000000E+00 0.00000000E+00 0.00000000E+00

0.00000000E+00 0.00000000E+00 0.00000000E+00

0.00000000E+00 0.00000000E+00 0.00000000E+00

0.00000000E+00 0.00000000E+00 0.00000000E+00

0.00000000E+00 0.00000000E+00 0.00000000E+00

0.00000000E+00 0.00000000E+00 0.00000000E+00

0.00000000E+00 0.00000000E+00 0.00000000E+00

0.00000000E+00 0.00000000E+00 0.00000000E+00

0.00000000E+00 0.00000000E+00 0.00000000E+00

0.00000000E+00 0.00000000E+00 0.00000000E+00

0.00000000E+00 0.00000000E+00 0.00000000E+00

0.00000000E+00 0.00000000E+00 0.00000000E+00

0.00000000E+00 0.00000000E+00 0.00000000E+00

0.00000000E+00 0.00000000E+00 0.00000000E+00

0.00000000E+00 0.00000000E+00 0.00000000E+00

0.00000000E+00 0.00000000E+00 0.00000000E+00

0.00000000E+00 0.00000000E+00 0.00000000E+00

0.00000000E+00 0.00000000E+00 0.00000000E+00

0.00000000E+00 0.00000000E+00 0.00000000E+00

0.00000000E+00 0.00000000E+00 0.00000000E+00

0.00000000E+00 0.00000000E+00 0.00000000E+00

0.00000000E+00 0.00000000E+00 0.00000000E+00

0.00000000E+00 0.00000000E+00 0.00000000E+00

0.00000000E+00 0.00000000E+00 0.00000000E+00

0.00000000E+00 0.00000000E+00 0.00000000E+00

0.00000000E+00 0.00000000E+00 0.00000000E+00

0.00000000E+00 0.00000000E+00 0.00000000E+00

0.00000000E+00 0.00000000E+00 0.00000000E+00

0.00000000E+00 0.00000000E+00 0.00000000E+00

0.00000000E+00 0.00000000E+00 0.00000000E+00

0.00000000E+00 0.00000000E+00 0.00000000E+00

0.00000000E+00 0.00000000E+00 0.00000000E+00

0.00000000E+00 0.00000000E+00 0.00000000E+00

0.00000000E+00 0.00000000E+00 0.00000000E+00

0.00000000E+00 0.00000000E+00 0.00000000E+00

0.00000000E+00 0.00000000E+00 0.00000000E+00

0.00000000E+00 0.00000000E+00 0.00000000E+00

0.00000000E+00 0.00000000E+00 0.00000000E+00

0.00000000E+00 0.00000000E+00 0.00000000E+00

0.00000000E+00 0.00000000E+00 0.00000000E+00

0.00000000E+00 0.00000000E+00 0.00000000E+00

0.00000000E+00 0.00000000E+00 0.00000000E+00

0.00000000E+00 0.00000000E+00 0.00000000E+00

0.00000000E+00 0.00000000E+00 0.00000000E+00

0.00000000E+00 0.00000000E+00 0.00000000E+00

0.00000000E+00 0.00000000E+00 0.00000000E+00

0.00000000E+00 0.00000000E+00 0.00000000E+00

0.00000000E+00 0.00000000E+00 0.00000000E+00

0.00000000E+00 0.00000000E+00 0.00000000E+00

0.00000000E+00 0.00000000E+00 0.00000000E+00

0.00000000E+00 0.00000000E+00 0.00000000E+00

0.00000000E+00 0.00000000E+00 0.00000000E+00

0.00000000E+00 0.00000000E+00 0.00000000E+00

0.00000000E+00 0.00000000E+00 0.00000000E+00

0.00000000E+00 0.00000000E+00 0.00000000E+00

0.00000000E+00 0.00000000E+00 0.00000000E+00

0.00000000E+00 0.00000000E+00 0.00000000E+00

0.00000000E+00 0.00000000E+00 0.00000000E+00

0.00000000E+00 0.00000000E+00 0.00000000E+00

0.00000000E+00 0.00000000E+00 0.00000000E+00

0.00000000E+00 0.00000000E+00 0.00000000E+00

0.00000000E+00 0.00000000E+00 0.00000000E+00

0.00000000E+00 0.00000000E+00 0.00000000E+00

0.00000000E+00 0.00000000E+00 0.00000000E+00

0.00000000E+00 0.00000000E+00 0.00000000E+00

0.00000000E+00 0.00000000E+00 0.00000000E+00

0.00000000E+00 0.00000000E+00 0.00000000E+00

0.00000000E+00 0.00000000E+00 0.00000000E+00

0.00000000E+00 0.00000000E+00 0.00000000E+00

0.00000000E+00 0.00000000E+00 0.00000000E+00

0.00000000E+00 0.00000000E+00 0.00000000E+00

0.00000000E+00 0.00000000E+00 0.00000000E+00

0.00000000E+00 0.00000000E+00 0.00000000E+00

0.00000000E+00 0.00000000E+00 0.00000000E+00

0.00000000E+00 0.00000000E+00 0.00000000E+00

0.00000000E+00 0.00000000E+00 0.00000000E+00

0.00000000E+00 0.00000000E+00 0.00000000E+00

0.00000000E+00 0.00000000E+00 0.00000000E+00

0.00000000E+00 0.00000000E+00 0.00000000E+00

0.00000000E+00 0.00000000E+00 0.00000000E+00

0.00000000E+00 0.00000000E+00 0.00000000E+00

0.00000000E+00 0.00000000E+00 0.00000000E+00

0.00000000E+00 0.00000000E+00 0.00000000E+00

0.00000000E+00 0.00000000E+00 0.00000000E+00

0.00000000E+00 0.00000000E+00 0.00000000E+00

0.00000000E+00 0.00000000E+00 0.00000000E+00
