# Supplementary material for: Boosted high-throughput D⁺ transfer from D₂O to unsaturated bonds via Pdδ+ cathode for solvent-free deuteration
Source: Nat Commun. 2025 May 15;16:4503. doi: 10.1038/s41467-025-59776-1 (PMC12081598; doi:10.1038/s41467-025-59776-1)
Supplement: Supplementary file 4 — Supplementary Data 2 [file 41467_2025_59776_MOESM4_ESM.docx]

N10C

1.00000000000000

17.2199993133999989 0.0000000000000000 0.0000000000000000

-8.6099996566999994 14.9129568584999994 0.0000000000000000

0.0000000000000000 0.0000000000000000 20.0000000000000000

C N Pd

92 3 14

Direct

0.9962588744381073 0.0014988943951989 0.0918104679766511

0.0444067737386081 0.0951891339519483 0.1022255459027115

0.1387675576938837 0.0005216014546258 0.0917380974813597

0.1872053048142680 0.0945983210784569 0.0998180372453107

0.2820182879936395 0.0017257779371028 0.0882640734332140

0.3314672559936904 0.0959681364899133 0.0931024624501078

0.4205416270402710 0.9924870739457970 0.0842695991801798

0.4708120041542164 0.0901039888593336 0.0856172783557074

0.5560015968695754 0.9859776844285479 0.0813054650372212

0.6069120402064853 0.0851367423784374 0.0811272268295637

0.7037704882871718 0.9954837261123808 0.0784205910390738

0.8545153900255779 0.0116957750328292 0.0852733840253635

0.9075219223806076 0.1146575575569046 0.0921555201389141

-0.0013117043784233 0.1448780927800508 0.1050089575818636

0.0507159390006822 0.2391267343166951 0.1174503897275473

0.1400395122793690 0.1418309522119098 0.1070493455503327

0.1899771018107764 0.2356563066099538 0.1174966529789482

0.2825462712708963 0.1412909331292961 0.1001019797774651

0.3304680835219019 0.2347339681465146 0.1072921571011472

0.4273646514741486 0.1429040806353129 0.0905380488101284

0.4768567975341452 0.2395272190290631 0.0931657304219316

0.5661388145658404 0.1400598275048711 0.0833605760403347

0.6180937891838713 0.2390218186501605 0.0828006363322067

0.7032535983789990 0.1300409196499123 0.0789892995871074

0.7593359647278922 0.2278871378658215 0.0803214908708140

0.9095658363239423 0.2212713957389968 0.0915590347204787

0.0009866864419123 0.2828624060719772 0.1046688424439808

0.0492557315185585 0.3790050144020167 0.1015426218470675

0.1444812423989874 0.2855281647453564 0.1253652915783149

0.1933606000762680 0.3830310128877022 0.1249486438908528

0.2840025290024250 0.2815373669935147 0.1171605770843548

0.3337794550306509 0.3773669275905036 0.1218323315553264

0.4257891340201642 0.2835316764547488 0.1039835756440458

0.5736044636102628 0.2947302343247958 0.0860886973746463

0.6275396484669781 0.3957064143581281 0.0830963118519857

0.7147815388506854 0.2805702370028019 0.0803918961070581

0.7653310286365126 0.3767678680299794 0.0790861597376002

0.8568809681290297 0.2693933283442987 0.0840203211171171

0.9074115677243970 0.3716764559455750 0.0830768010823130

0.0039233341496277 0.4266382728758163 0.0884202121045722

0.0560369702171212 0.5258095818669104 0.0853566459887871

0.1446609897614657 0.4295820971010532 0.1109597102642353

0.1955566139665547 0.5254715044575170 0.1008744150777421

0.2888140186340858 0.4283171334805670 0.1187341152532018

0.3393278366576946 0.5213420890282600 0.1051912646458249

0.4292813155015727 0.4200627954217240 0.1130890845762051

0.4813791975496088 0.5146572471502304 0.0999806194074879

0.6284647654176374 0.5067357654336346 0.0831377317668778

0.7206837197518940 0.4283961804682170 0.0789916129913984

0.7728506293846139 0.5236263300180671 0.0766487035004953

0.8615245503128548 0.4226737448944659 0.0789704738191836

0.9120425070094959 0.5206388833484057 0.0776124432894979

0.0074744949993748 0.5716608746578130 0.0796356818735934

0.0556768872805637 0.6687460791803299 0.0791339039907544

0.1541191858642104 0.5784919932821958 0.0898596645010285

0.2081099187135693 0.6792786854902407 0.0857029962294883

0.2914789533431827 0.5704273968500981 0.1019676821799260

0.3403262358328995 0.6651392818638304 0.0944097871618737

0.4345835974747943 0.5634828248914326 0.0972572285465152

0.4814374754930055 0.6584162277563598 0.0890268717070485

0.5765737609716703 0.5548275125624508 0.0881235091719452

0.6226148498138572 0.6553879263939530 0.0832421884339683

0.7212106802224807 0.5670227790887398 0.0782292094203480

0.7659057244600428 0.6634061799367781 0.0779696861523072

0.8656800154236665 0.5704912634082090 0.0759832706040328

0.9108906147963838 0.6670847093814197 0.0766089448998469

0.0052723465658225 0.7155378452456145 0.0780747116909469

0.0477152141890123 0.8108141097761765 0.0807966516958633

0.1513831661573621 0.7190961349778004 0.0810628268073783

0.1924418793363271 0.8153830102228518 0.0810117713561558

0.3093923934374512 0.7281127606073852 0.0885499879092838

0.4343234785769443 0.7050616247558906 0.0895615155857011

0.4728850168454155 0.7985385653357995 0.0843638563103478

0.5758597719852063 0.7059232412736127 0.0840128933058428

0.6187767644201831 0.8030188234023231 0.0812689285652610

0.7170999075668592 0.7098313170690541 0.0796500991902529

0.7617481016185694 0.8078499090196304 0.0799158280985762

0.8609668647682496 0.7139665547151677 0.0775164878595719

0.9044788127397666 0.8105585871848152 0.0801749542319285

0.9989028998665666 0.8579535692615362 0.0825337499459721

0.0440706336633366 0.9530404493170218 0.0885544233891740

0.1416755222130945 0.8598568438601064 0.0827301292929750

0.1873550289237736 0.9542578779668547 0.0873158131976948

0.3254190811254742 0.9506959616735130 0.0841236815948685

0.4093210357180169 0.8293152479643553 0.0844904985541784

0.4570051307839776 0.9322916758741591 0.0831456301900452

0.5672657977509307 0.8482752658125963 0.0815782487835010

0.6086030055954023 0.9429884246947546 0.0801329715422994

0.7143622927839596 0.8565468475405288 0.0801425769980574

0.7586342184181970 0.9538336083828420 0.0803133947895485

0.8562727934420670 0.8598961679468163 0.0814421150371585

0.9013690150618501 0.9583801475403132 0.0859808068539736

0.4712243410343163 0.3723508642221414 0.1090574830711973

0.2810737725199096 0.8616996917808047 0.0807623865686048

0.7469873797924997 0.0845689800892722 0.0763797426038559

0.0897586280494993 0.2414219239437080 0.2268338210078749

0.0762095729608839 0.3516502154402757 0.3122743417973163

0.2457182801542127 0.2692062607379118 0.2806439130465789

0.2327343125962885 0.4125507225631517 0.2336267395330684

0.3884452411527842 0.4053922677116057 0.2244097129527723

0.3800061187217112 0.5248276167519619 0.3079758411482616

0.1189189829485654 0.4743913985277089 0.2218479382205510

0.2212966149138039 0.5428628323945702 0.3261265351799336

0.3194075705594648 0.5951823797885608 0.2198397949071520

0.1043039391775167 0.2225693832247408 0.3543600453168133

0.2352157773976003 0.3856562831586575 0.3713009782323675

0.3828445907658939 0.3819926521496323 0.3528425950247260

0.1348176006552574 0.4405333031001230 0.4242557044826979

0.3185298368050959 0.5435723536634991 0.4236860456610642

0.00000000E+00 0.00000000E+00 0.00000000E+00

0.00000000E+00 0.00000000E+00 0.00000000E+00

0.00000000E+00 0.00000000E+00 0.00000000E+00

0.00000000E+00 0.00000000E+00 0.00000000E+00

0.00000000E+00 0.00000000E+00 0.00000000E+00

0.00000000E+00 0.00000000E+00 0.00000000E+00

0.00000000E+00 0.00000000E+00 0.00000000E+00

0.00000000E+00 0.00000000E+00 0.00000000E+00

0.00000000E+00 0.00000000E+00 0.00000000E+00

0.00000000E+00 0.00000000E+00 0.00000000E+00

0.00000000E+00 0.00000000E+00 0.00000000E+00

0.00000000E+00 0.00000000E+00 0.00000000E+00

0.00000000E+00 0.00000000E+00 0.00000000E+00

0.00000000E+00 0.00000000E+00 0.00000000E+00

0.00000000E+00 0.00000000E+00 0.00000000E+00

0.00000000E+00 0.00000000E+00 0.00000000E+00

0.00000000E+00 0.00000000E+00 0.00000000E+00

0.00000000E+00 0.00000000E+00 0.00000000E+00

0.00000000E+00 0.00000000E+00 0.00000000E+00

0.00000000E+00 0.00000000E+00 0.00000000E+00

0.00000000E+00 0.00000000E+00 0.00000000E+00

0.00000000E+00 0.00000000E+00 0.00000000E+00

0.00000000E+00 0.00000000E+00 0.00000000E+00

0.00000000E+00 0.00000000E+00 0.00000000E+00

0.00000000E+00 0.00000000E+00 0.00000000E+00

0.00000000E+00 0.00000000E+00 0.00000000E+00

0.00000000E+00 0.00000000E+00 0.00000000E+00

0.00000000E+00 0.00000000E+00 0.00000000E+00

0.00000000E+00 0.00000000E+00 0.00000000E+00

0.00000000E+00 0.00000000E+00 0.00000000E+00

0.00000000E+00 0.00000000E+00 0.00000000E+00

0.00000000E+00 0.00000000E+00 0.00000000E+00

0.00000000E+00 0.00000000E+00 0.00000000E+00

0.00000000E+00 0.00000000E+00 0.00000000E+00

0.00000000E+00 0.00000000E+00 0.00000000E+00

0.00000000E+00 0.00000000E+00 0.00000000E+00

0.00000000E+00 0.00000000E+00 0.00000000E+00

0.00000000E+00 0.00000000E+00 0.00000000E+00

0.00000000E+00 0.00000000E+00 0.00000000E+00

0.00000000E+00 0.00000000E+00 0.00000000E+00

0.00000000E+00 0.00000000E+00 0.00000000E+00

0.00000000E+00 0.00000000E+00 0.00000000E+00

0.00000000E+00 0.00000000E+00 0.00000000E+00

0.00000000E+00 0.00000000E+00 0.00000000E+00

0.00000000E+00 0.00000000E+00 0.00000000E+00

0.00000000E+00 0.00000000E+00 0.00000000E+00

0.00000000E+00 0.00000000E+00 0.00000000E+00

0.00000000E+00 0.00000000E+00 0.00000000E+00

0.00000000E+00 0.00000000E+00 0.00000000E+00

0.00000000E+00 0.00000000E+00 0.00000000E+00

0.00000000E+00 0.00000000E+00 0.00000000E+00

0.00000000E+00 0.00000000E+00 0.00000000E+00

0.00000000E+00 0.00000000E+00 0.00000000E+00

0.00000000E+00 0.00000000E+00 0.00000000E+00

0.00000000E+00 0.00000000E+00 0.00000000E+00

0.00000000E+00 0.00000000E+00 0.00000000E+00

0.00000000E+00 0.00000000E+00 0.00000000E+00

0.00000000E+00 0.00000000E+00 0.00000000E+00

0.00000000E+00 0.00000000E+00 0.00000000E+00

0.00000000E+00 0.00000000E+00 0.00000000E+00

0.00000000E+00 0.00000000E+00 0.00000000E+00

0.00000000E+00 0.00000000E+00 0.00000000E+00

0.00000000E+00 0.00000000E+00 0.00000000E+00

0.00000000E+00 0.00000000E+00 0.00000000E+00

0.00000000E+00 0.00000000E+00 0.00000000E+00

0.00000000E+00 0.00000000E+00 0.00000000E+00

0.00000000E+00 0.00000000E+00 0.00000000E+00

0.00000000E+00 0.00000000E+00 0.00000000E+00

0.00000000E+00 0.00000000E+00 0.00000000E+00

0.00000000E+00 0.00000000E+00 0.00000000E+00

0.00000000E+00 0.00000000E+00 0.00000000E+00

0.00000000E+00 0.00000000E+00 0.00000000E+00

0.00000000E+00 0.00000000E+00 0.00000000E+00

0.00000000E+00 0.00000000E+00 0.00000000E+00

0.00000000E+00 0.00000000E+00 0.00000000E+00

0.00000000E+00 0.00000000E+00 0.00000000E+00

0.00000000E+00 0.00000000E+00 0.00000000E+00

0.00000000E+00 0.00000000E+00 0.00000000E+00

0.00000000E+00 0.00000000E+00 0.00000000E+00

0.00000000E+00 0.00000000E+00 0.00000000E+00

0.00000000E+00 0.00000000E+00 0.00000000E+00

0.00000000E+00 0.00000000E+00 0.00000000E+00

0.00000000E+00 0.00000000E+00 0.00000000E+00

0.00000000E+00 0.00000000E+00 0.00000000E+00

0.00000000E+00 0.00000000E+00 0.00000000E+00

0.00000000E+00 0.00000000E+00 0.00000000E+00

0.00000000E+00 0.00000000E+00 0.00000000E+00

0.00000000E+00 0.00000000E+00 0.00000000E+00

0.00000000E+00 0.00000000E+00 0.00000000E+00

0.00000000E+00 0.00000000E+00 0.00000000E+00

0.00000000E+00 0.00000000E+00 0.00000000E+00

0.00000000E+00 0.00000000E+00 0.00000000E+00

0.00000000E+00 0.00000000E+00 0.00000000E+00

0.00000000E+00 0.00000000E+00 0.00000000E+00

0.00000000E+00 0.00000000E+00 0.00000000E+00

0.00000000E+00 0.00000000E+00 0.00000000E+00

0.00000000E+00 0.00000000E+00 0.00000000E+00

0.00000000E+00 0.00000000E+00 0.00000000E+00

0.00000000E+00 0.00000000E+00 0.00000000E+00

0.00000000E+00 0.00000000E+00 0.00000000E+00

0.00000000E+00 0.00000000E+00 0.00000000E+00

0.00000000E+00 0.00000000E+00 0.00000000E+00

0.00000000E+00 0.00000000E+00 0.00000000E+00

0.00000000E+00 0.00000000E+00 0.00000000E+00

0.00000000E+00 0.00000000E+00 0.00000000E+00

0.00000000E+00 0.00000000E+00 0.00000000E+00

0.00000000E+00 0.00000000E+00 0.00000000E+00

0.00000000E+00 0.00000000E+00 0.00000000E+00

0.00000000E+00 0.00000000E+00 0.00000000E+00
