# Supplementary material for: Boosted high-throughput D⁺ transfer from D₂O to unsaturated bonds via Pdδ+ cathode for solvent-free deuteration
Source: Nat Commun. 2025 May 15;16:4503. doi: 10.1038/s41467-025-59776-1 (PMC12081598; doi:10.1038/s41467-025-59776-1)
Supplement: Supplementary file 5 — Supplementary Data 3 [file 41467_2025_59776_MOESM5_ESM.docx]

Pd\(1\1\1)\(2)

1.00000000000000

11.0045995712000000 0.0000000000000000 0.0000000000000000

-5.5022997856000000 9.5302627871999999 0.0000000000000000

0.0000000000000000 0.0000000000000000 19.4925994873000015

Pd

48

Selective dynamics

Direct

0.0000017452937873 0.9999955230807881 0.3344767373869946 T T T

0.1666699949999995 0.0833299980000035 0.2178400009999990 F F F

0.0833299980000035 0.1666699949999995 0.1026000009999990 F F F

0.2500039270460853 0.9999973226223737 0.3344824521331307 T T T

0.4166699949999995 0.0833299980000035 0.2178400009999990 F F F

0.3333300050000005 0.1666699949999995 0.1026000009999990 F F F

0.5000037922086444 0.9999974193008597 0.3344860420555780 T T T

0.6666700239999983 0.0833299980000035 0.2178400009999990 F F F

0.5833299760000017 0.1666699949999995 0.1026000009999990 F F F

0.7500025970672434 0.9999974657489178 0.3344836411124407 T T T

0.9166700239999983 0.0833299980000035 0.2178400009999990 F F F

0.8333299760000017 0.1666699949999995 0.1026000009999990 F F F

0.0000018088331687 0.2499963253475838 0.3344786056055284 T T T

0.1666699949999995 0.3333300050000005 0.2178400009999990 F F F

0.0833299980000035 0.4166699949999995 0.1026000009999990 F F F

0.2500034828485573 0.2499959710795066 0.3344801607598811 T T T

0.4166699949999995 0.3333300050000005 0.2178400009999990 F F F

0.3333300050000005 0.4166699949999995 0.1026000009999990 F F F

0.5000039545134239 0.2499971007264414 0.3344799655382659 T T T

0.6666700239999983 0.3333300050000005 0.2178400009999990 F F F

0.5833299760000017 0.4166699949999995 0.1026000009999990 F F F

0.7500021268809074 0.2499969363959116 0.3344843628488418 T T T

0.9166700239999983 0.3333300050000005 0.2178400009999990 F F F

0.8333299760000017 0.4166699949999995 0.1026000009999990 F F F

0.0000015290256167 0.4999971194125827 0.3344780243278593 T T T

0.1666699949999995 0.5833299760000017 0.2178400009999990 F F F

0.0833299980000035 0.6666700239999983 0.1026000009999990 F F F

0.2500025974548271 0.4999970512649312 0.3344873249529702 T T T

0.4166699949999995 0.5833299760000017 0.2178400009999990 F F F

0.3333300050000005 0.6666700239999983 0.1026000009999990 F F F

0.5000050466451808 0.4999976816748963 0.3344810977937722 T T T

0.6666700239999983 0.5833299760000017 0.2178400009999990 F F F

0.5833299760000017 0.6666700239999983 0.1026000009999990 F F F

0.7500028509098180 0.4999957383659907 0.3344840762680068 T T T

0.9166700239999983 0.5833299760000017 0.2178400009999990 F F F

0.8333299760000017 0.6666700239999983 0.1026000009999990 F F F

0.0000023449624366 0.7499962518089079 0.3344787364929087 T T T

0.1666699949999995 0.8333299760000017 0.2178400009999990 F F F

0.0833299980000035 0.9166700239999983 0.1026000009999990 F F F

0.2500029123186118 0.7499976400191956 0.3344862710758429 T T T

0.4166699949999995 0.8333299760000017 0.2178400009999990 F F F

0.3333300050000005 0.9166700239999983 0.1026000009999990 F F F

0.5000039414641844 0.7499976812877667 0.3344841993457464 T T T

0.6666700239999983 0.8333299760000017 0.2178400009999990 F F F

0.5833299760000017 0.9166700239999983 0.1026000009999990 F F F

0.7500032246496947 0.7499967688607256 0.3344804327938896 T T T

0.9166700239999983 0.8333299760000017 0.2178400009999990 F F F

0.8333299760000017 0.9166700239999983 0.1026000009999990 F F F

0.00000000E+00 0.00000000E+00 0.00000000E+00

0.00000000E+00 0.00000000E+00 0.00000000E+00

0.00000000E+00 0.00000000E+00 0.00000000E+00

0.00000000E+00 0.00000000E+00 0.00000000E+00

0.00000000E+00 0.00000000E+00 0.00000000E+00

0.00000000E+00 0.00000000E+00 0.00000000E+00

0.00000000E+00 0.00000000E+00 0.00000000E+00

0.00000000E+00 0.00000000E+00 0.00000000E+00

0.00000000E+00 0.00000000E+00 0.00000000E+00

0.00000000E+00 0.00000000E+00 0.00000000E+00

0.00000000E+00 0.00000000E+00 0.00000000E+00

0.00000000E+00 0.00000000E+00 0.00000000E+00

0.00000000E+00 0.00000000E+00 0.00000000E+00

0.00000000E+00 0.00000000E+00 0.00000000E+00

0.00000000E+00 0.00000000E+00 0.00000000E+00

0.00000000E+00 0.00000000E+00 0.00000000E+00

0.00000000E+00 0.00000000E+00 0.00000000E+00

0.00000000E+00 0.00000000E+00 0.00000000E+00

0.00000000E+00 0.00000000E+00 0.00000000E+00

0.00000000E+00 0.00000000E+00 0.00000000E+00

0.00000000E+00 0.00000000E+00 0.00000000E+00

0.00000000E+00 0.00000000E+00 0.00000000E+00

0.00000000E+00 0.00000000E+00 0.00000000E+00

0.00000000E+00 0.00000000E+00 0.00000000E+00

0.00000000E+00 0.00000000E+00 0.00000000E+00

0.00000000E+00 0.00000000E+00 0.00000000E+00

0.00000000E+00 0.00000000E+00 0.00000000E+00

0.00000000E+00 0.00000000E+00 0.00000000E+00

0.00000000E+00 0.00000000E+00 0.00000000E+00

0.00000000E+00 0.00000000E+00 0.00000000E+00

0.00000000E+00 0.00000000E+00 0.00000000E+00

0.00000000E+00 0.00000000E+00 0.00000000E+00

0.00000000E+00 0.00000000E+00 0.00000000E+00

0.00000000E+00 0.00000000E+00 0.00000000E+00

0.00000000E+00 0.00000000E+00 0.00000000E+00

0.00000000E+00 0.00000000E+00 0.00000000E+00

0.00000000E+00 0.00000000E+00 0.00000000E+00

0.00000000E+00 0.00000000E+00 0.00000000E+00

0.00000000E+00 0.00000000E+00 0.00000000E+00

0.00000000E+00 0.00000000E+00 0.00000000E+00

0.00000000E+00 0.00000000E+00 0.00000000E+00

0.00000000E+00 0.00000000E+00 0.00000000E+00

0.00000000E+00 0.00000000E+00 0.00000000E+00

0.00000000E+00 0.00000000E+00 0.00000000E+00

0.00000000E+00 0.00000000E+00 0.00000000E+00

0.00000000E+00 0.00000000E+00 0.00000000E+00

0.00000000E+00 0.00000000E+00 0.00000000E+00

0.00000000E+00 0.00000000E+00 0.00000000E+00
