# Supplementary material for: Boosted high-throughput D⁺ transfer from D₂O to unsaturated bonds via Pdδ+ cathode for solvent-free deuteration
Source: Nat Commun. 2025 May 15;16:4503. doi: 10.1038/s41467-025-59776-1 (PMC12081598; doi:10.1038/s41467-025-59776-1)
Supplement: Supplementary file 6 — Supplementary Data 4 [file 41467_2025_59776_MOESM6_ESM.docx]

Pd\(1\1\1)\(2)

1.00000000000000

11.0045995712000000 0.0000000000000000 0.0000000000000000

-5.5022997856000000 9.5302627871999999 0.0000000000000000

0.0000000000000000 0.0000000000000000 19.4925994873000015

Pd

48

Selective dynamics

Direct

0.0000013226627897 0.9999953678726032 0.3345115449671789 T T T

0.1666699949999995 0.0833299980000035 0.2178400009999990 F F F

0.0833299980000035 0.1666699949999995 0.1026000009999990 F F F

0.2500035094148835 0.9999974353497950 0.3345171600565044 T T T

0.4166699949999995 0.0833299980000035 0.2178400009999990 F F F

0.3333300050000005 0.1666699949999995 0.1026000009999990 F F F

0.5000032630241620 0.9999979608499979 0.3345216404292112 T T T

0.6666700239999983 0.0833299980000035 0.2178400009999990 F F F

0.5833299760000017 0.1666699949999995 0.1026000009999990 F F F

0.7500024786844183 0.9999982323507426 0.3345195168785029 T T T

0.9166700239999983 0.0833299980000035 0.2178400009999990 F F F

0.8333299760000017 0.1666699949999995 0.1026000009999990 F F F

0.0000010105359698 0.2499969253302086 0.3345133955450754 T T T

0.1666699949999995 0.3333300050000005 0.2178400009999990 F F F

0.0833299980000035 0.4166699949999995 0.1026000009999990 F F F

0.2500031257770198 0.2499957786336791 0.3345150686807501 T T T

0.4166699949999995 0.3333300050000005 0.2178400009999990 F F F

0.3333300050000005 0.4166699949999995 0.1026000009999990 F F F

0.5000035799829164 0.2499972093826856 0.3345146936044336 T T T

0.6666700239999983 0.3333300050000005 0.2178400009999990 F F F

0.5833299760000017 0.4166699949999995 0.1026000009999990 F F F

0.7500022276133684 0.2499977889719415 0.3345196410709195 T T T

0.9166700239999983 0.3333300050000005 0.2178400009999990 F F F

0.8333299760000017 0.4166699949999995 0.1026000009999990 F F F

0.0000008466730718 0.4999977210014380 0.3345121322526172 T T T

0.1666699949999995 0.5833299760000017 0.2178400009999990 F F F

0.0833299980000035 0.6666700239999983 0.1026000009999990 F F F

0.2500014239541443 0.4999973273135719 0.3345228819242499 T T T

0.4166699949999995 0.5833299760000017 0.2178400009999990 F F F

0.3333300050000005 0.6666700239999983 0.1026000009999990 F F F

0.5000051578512049 0.4999980303893978 0.3345164398058513 T T T

0.6666700239999983 0.5833299760000017 0.2178400009999990 F F F

0.5833299760000017 0.6666700239999983 0.1026000009999990 F F F

0.7500032845201712 0.4999961133482235 0.3345191910111786 T T T

0.9166700239999983 0.5833299760000017 0.2178400009999990 F F F

0.8333299760000017 0.6666700239999983 0.1026000009999990 F F F

0.0000021092611536 0.7499962468783391 0.3345129389708405 T T T

0.1666699949999995 0.8333299760000017 0.2178400009999990 F F F

0.0833299980000035 0.9166700239999983 0.1026000009999990 F F F

0.2500016885742865 0.7499979699394443 0.3345214074619074 T T T

0.4166699949999995 0.8333299760000017 0.2178400009999990 F F F

0.3333300050000005 0.9166700239999983 0.1026000009999990 F F F

0.5000037698716261 0.7499983089586871 0.3345200580945116 T T T

0.6666700239999983 0.8333299760000017 0.2178400009999990 F F F

0.5833299760000017 0.9166700239999983 0.1026000009999990 F F F

0.7500034391038177 0.7499972055156962 0.3345157932934630 T T T

0.9166700239999983 0.8333299760000017 0.2178400009999990 F F F

0.8333299760000017 0.9166700239999983 0.1026000009999990 F F F

0.00000000E+00 0.00000000E+00 0.00000000E+00

0.00000000E+00 0.00000000E+00 0.00000000E+00

0.00000000E+00 0.00000000E+00 0.00000000E+00

0.00000000E+00 0.00000000E+00 0.00000000E+00

0.00000000E+00 0.00000000E+00 0.00000000E+00

0.00000000E+00 0.00000000E+00 0.00000000E+00

0.00000000E+00 0.00000000E+00 0.00000000E+00

0.00000000E+00 0.00000000E+00 0.00000000E+00

0.00000000E+00 0.00000000E+00 0.00000000E+00

0.00000000E+00 0.00000000E+00 0.00000000E+00

0.00000000E+00 0.00000000E+00 0.00000000E+00

0.00000000E+00 0.00000000E+00 0.00000000E+00

0.00000000E+00 0.00000000E+00 0.00000000E+00

0.00000000E+00 0.00000000E+00 0.00000000E+00

0.00000000E+00 0.00000000E+00 0.00000000E+00

0.00000000E+00 0.00000000E+00 0.00000000E+00

0.00000000E+00 0.00000000E+00 0.00000000E+00

0.00000000E+00 0.00000000E+00 0.00000000E+00

0.00000000E+00 0.00000000E+00 0.00000000E+00

0.00000000E+00 0.00000000E+00 0.00000000E+00

0.00000000E+00 0.00000000E+00 0.00000000E+00

0.00000000E+00 0.00000000E+00 0.00000000E+00

0.00000000E+00 0.00000000E+00 0.00000000E+00

0.00000000E+00 0.00000000E+00 0.00000000E+00

0.00000000E+00 0.00000000E+00 0.00000000E+00

0.00000000E+00 0.00000000E+00 0.00000000E+00

0.00000000E+00 0.00000000E+00 0.00000000E+00

0.00000000E+00 0.00000000E+00 0.00000000E+00

0.00000000E+00 0.00000000E+00 0.00000000E+00

0.00000000E+00 0.00000000E+00 0.00000000E+00

0.00000000E+00 0.00000000E+00 0.00000000E+00

0.00000000E+00 0.00000000E+00 0.00000000E+00

0.00000000E+00 0.00000000E+00 0.00000000E+00

0.00000000E+00 0.00000000E+00 0.00000000E+00

0.00000000E+00 0.00000000E+00 0.00000000E+00

0.00000000E+00 0.00000000E+00 0.00000000E+00

0.00000000E+00 0.00000000E+00 0.00000000E+00

0.00000000E+00 0.00000000E+00 0.00000000E+00

0.00000000E+00 0.00000000E+00 0.00000000E+00

0.00000000E+00 0.00000000E+00 0.00000000E+00

0.00000000E+00 0.00000000E+00 0.00000000E+00

0.00000000E+00 0.00000000E+00 0.00000000E+00

0.00000000E+00 0.00000000E+00 0.00000000E+00

0.00000000E+00 0.00000000E+00 0.00000000E+00

0.00000000E+00 0.00000000E+00 0.00000000E+00

0.00000000E+00 0.00000000E+00 0.00000000E+00

0.00000000E+00 0.00000000E+00 0.00000000E+00

0.00000000E+00 0.00000000E+00 0.00000000E+00
